# Supplementary material for: SERBP1 interacts with PARP1 and is present in PARylation-dependent protein complexes regulating splicing, cell division, and ribosome biogenesis
Source: eLife. 2025 Feb 12;13:RP98152. doi: 10.7554/eLife.98152 (PMC11820137; doi:10.7554/eLife.98152)
Supplement: Figure 1—figure supplement 1—source data 1. [file elife-98152-fig1-figsupp1-data1.pdf]

Figure 1–figure supplement 1 source data 1. PDF containing original protein gels for Figure 1–figure supplement 1.

Sonication Extract (M2021-007)

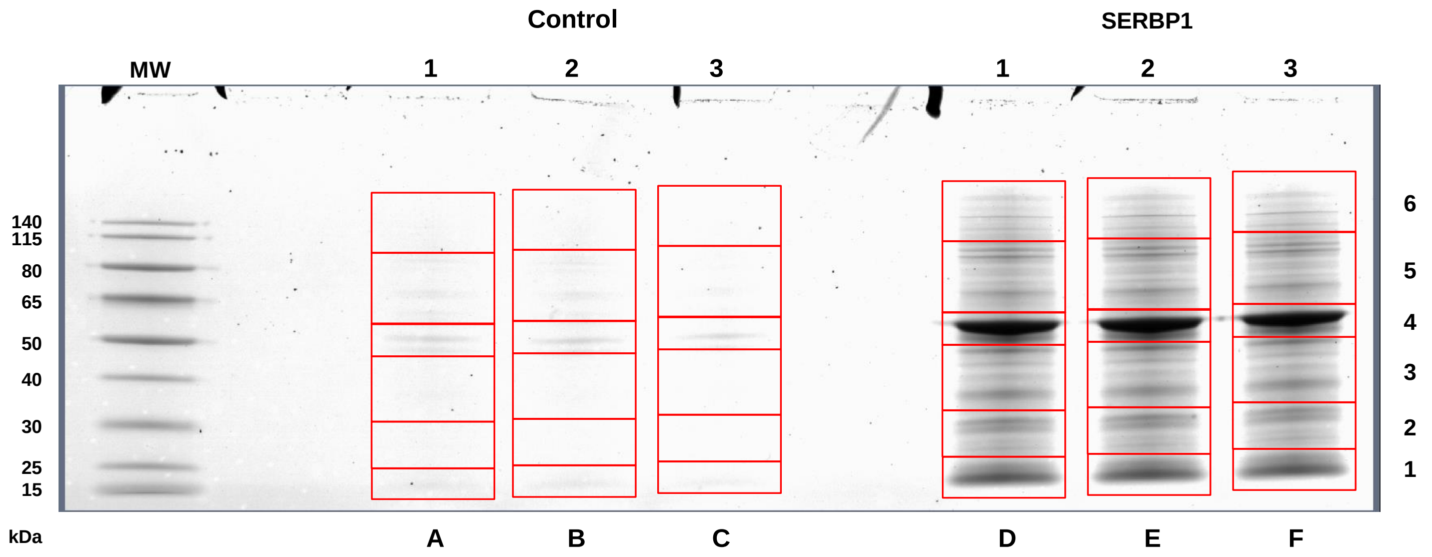

BioRad Criterion XT MOPS 12% SDS-PAGE reducing gel, colloidal Coomassie blue

Polysomal Lysis Buffer (M2021-065)

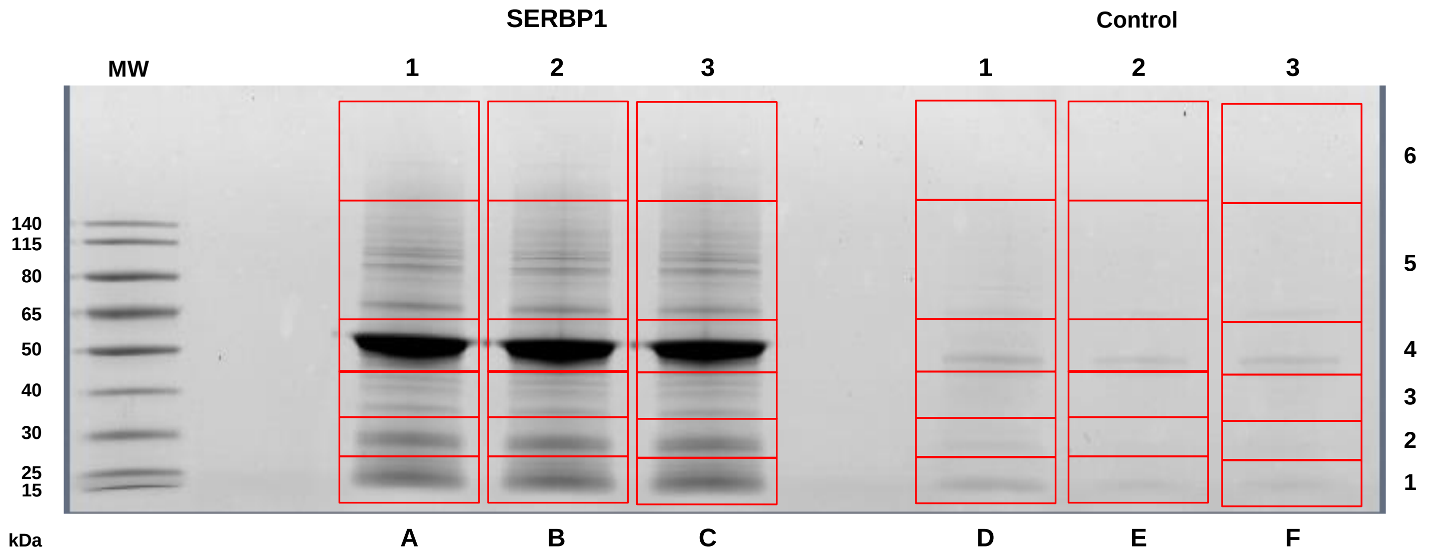

BioRad Criterion XT MOPS 12% SDS-PAGE reducing gel, colloidal Coomassie blue
