# Supplementary figures and images for: SERBP1 interacts with PARP1 and is present in PARylation-dependent protein complexes regulating splicing, cell division, and ribosome biogenesis
Source: eLife. 2025 Feb 12;13:RP98152. doi: 10.7554/eLife.98152 (PMC11820137; doi:10.7554/eLife.98152)

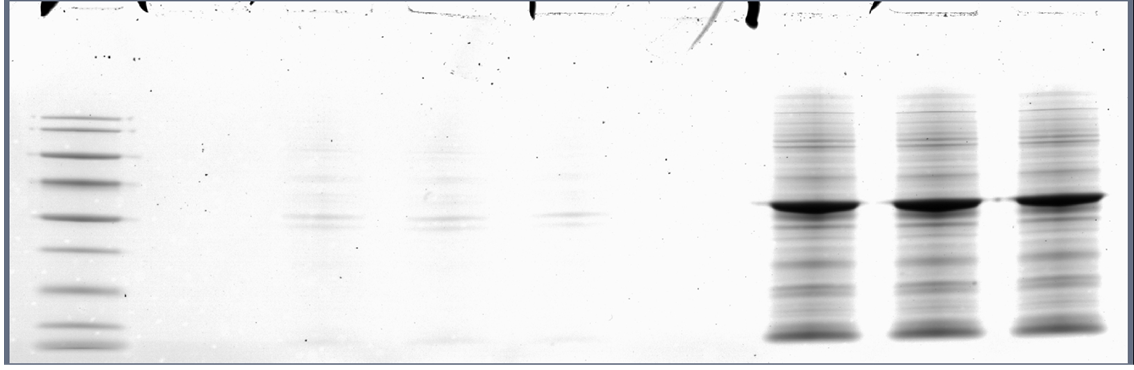

Supplement: Figure 1—figure supplement 1—source data 2. [file elife-98152-fig1-figsupp1-data2.zip › Figure 1-figure supplement 1 souce data 2. Original gels/Protein gel 1.tif]

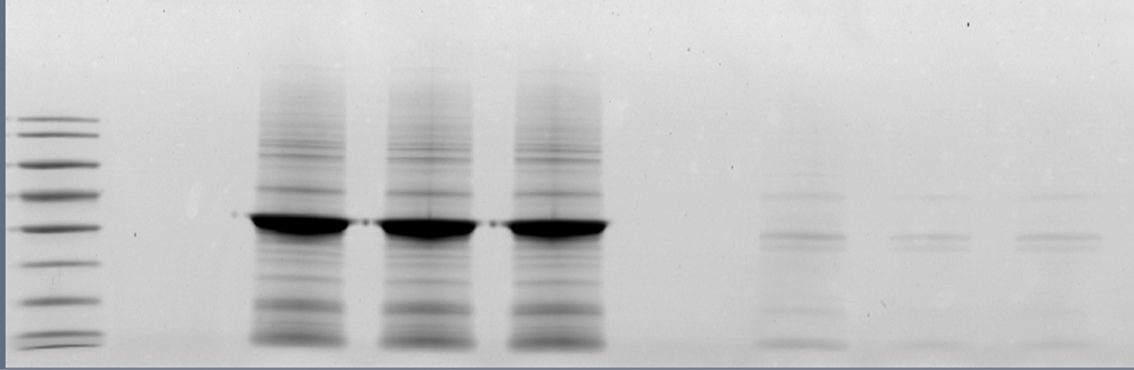

Supplement: Figure 1—figure supplement 1—source data 2. [file elife-98152-fig1-figsupp1-data2.zip › Figure 1-figure supplement 1 souce data 2. Original gels/Protein gel 2.tif]

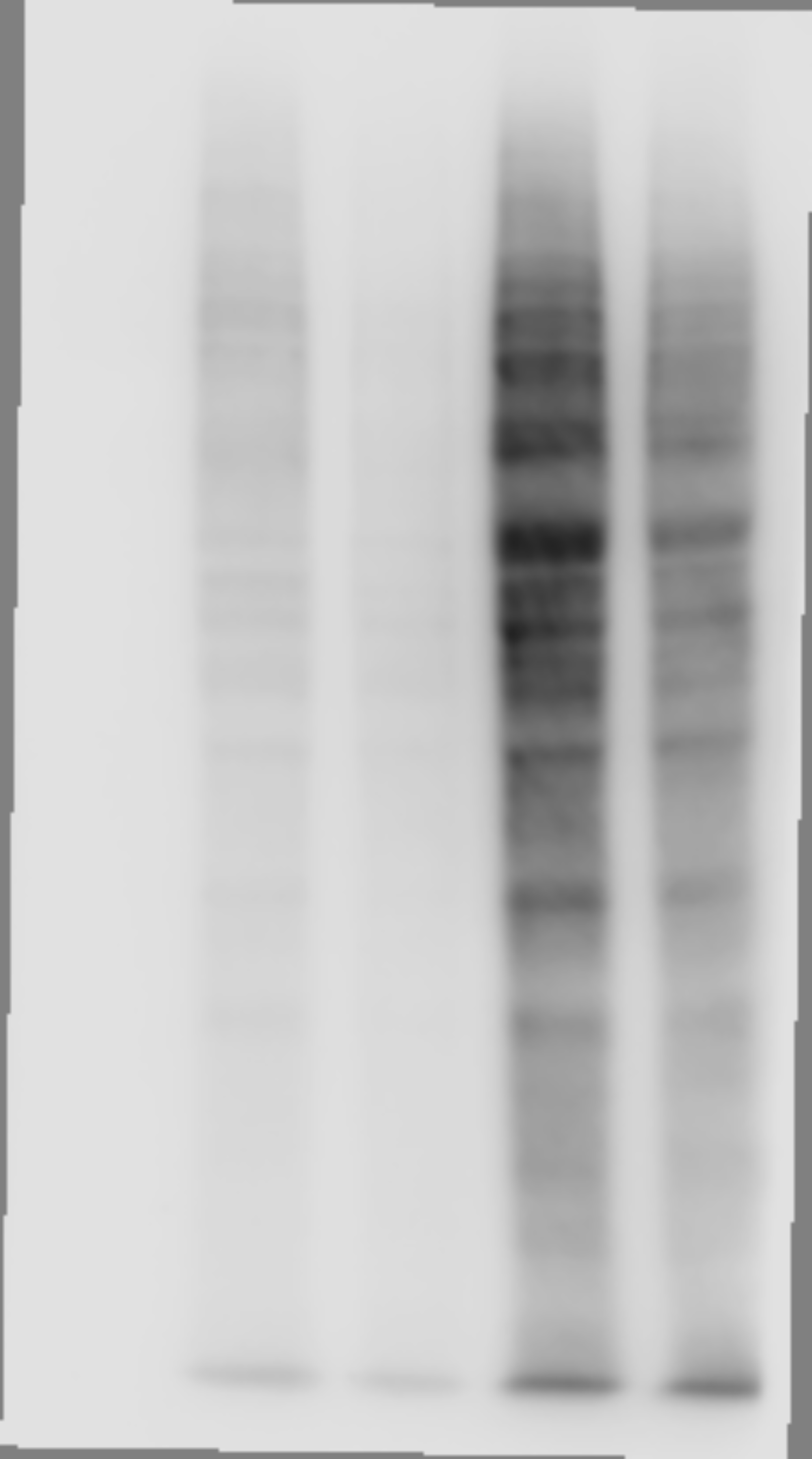

Supplement: Figure 1—figure supplement 2—source data 2. [file elife-98152-fig1-figsupp2-data2.zip › Figure 1-figure supplement 2. source data 2. Original westerns part A/S2A pur.tiff]

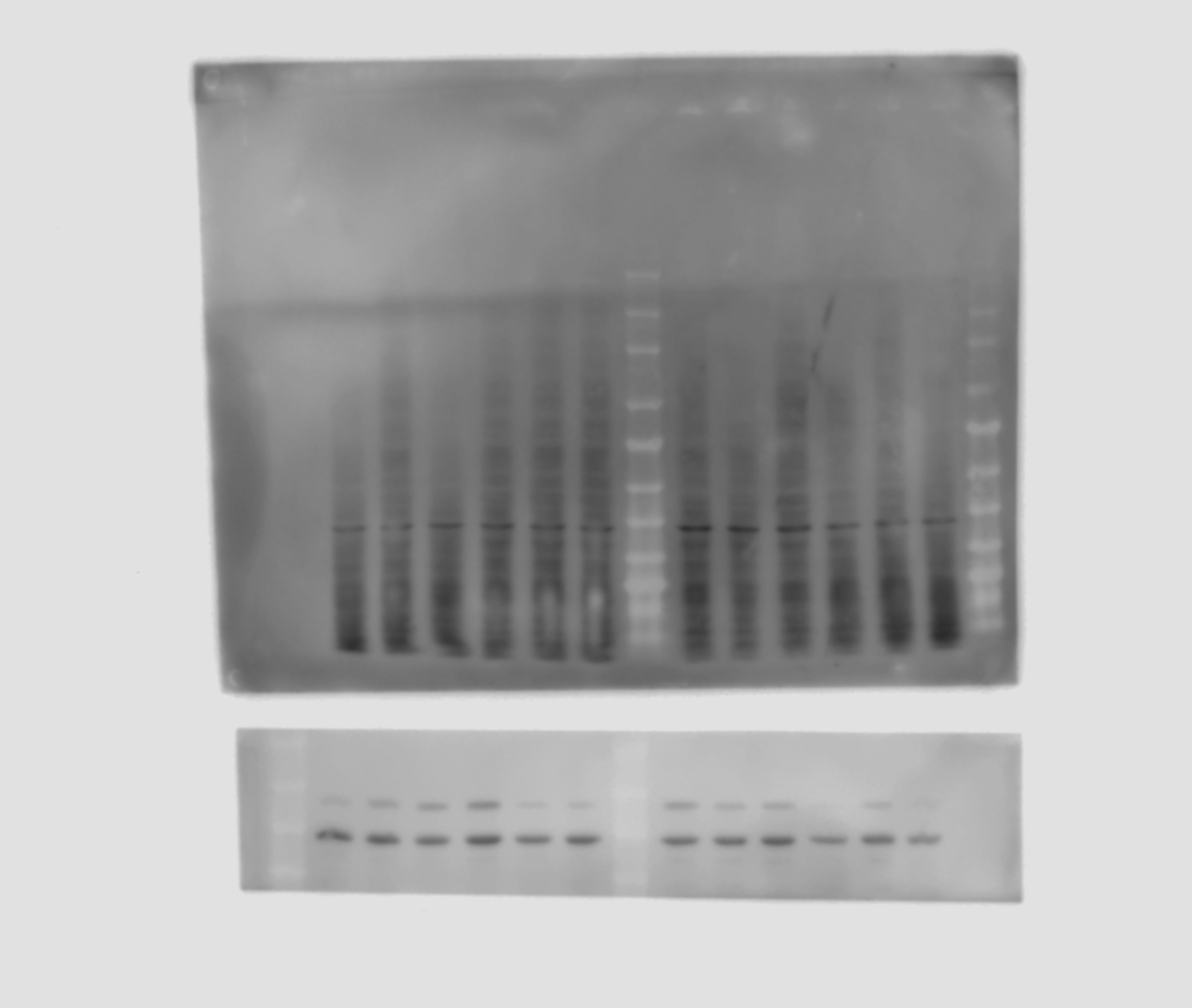

Supplement: Figure 1—figure supplement 2—source data 2. [file elife-98152-fig1-figsupp2-data2.zip › Figure 1-figure supplement 2. source data 2. Original westerns part A/S2A Actin.tif]

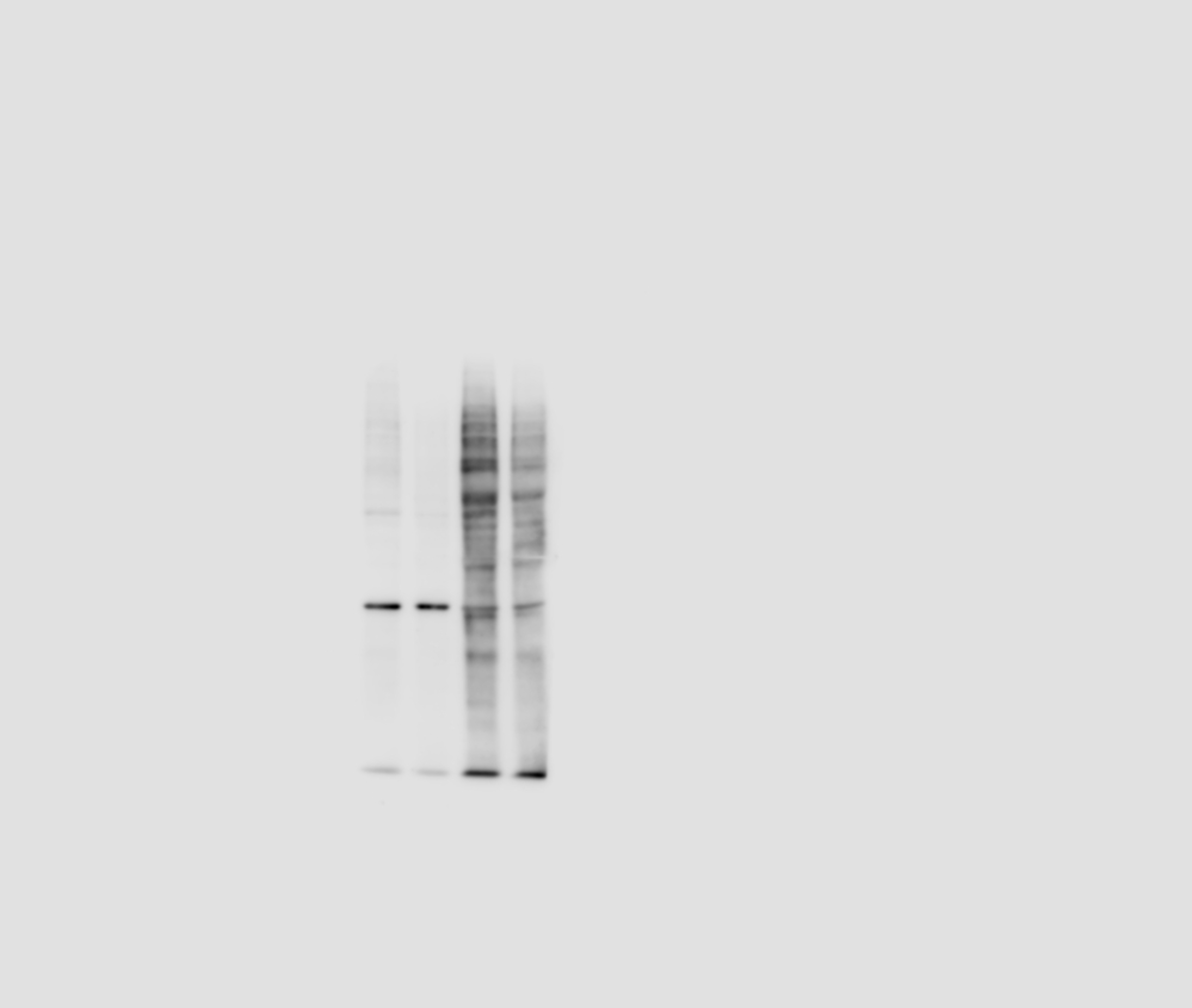

Supplement: Figure 1—figure supplement 2—source data 2. [file elife-98152-fig1-figsupp2-data2.zip › Figure 1-figure supplement 2. source data 2. Original westerns part A/S2A GAPDH.tif]

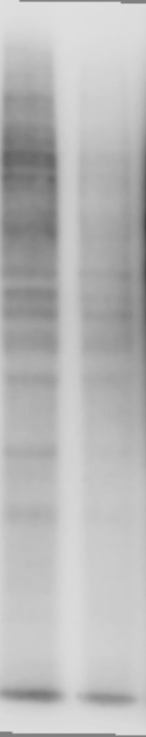

Supplement: Figure 1—figure supplement 2—source data 2. [file elife-98152-fig1-figsupp2-data2.zip › Figure 1-figure supplement 2. source data 2. Original westerns part A/S2A Puro U251.tif]

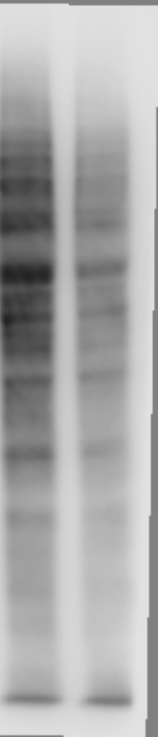

Supplement: Figure 1—figure supplement 2—source data 2. [file elife-98152-fig1-figsupp2-data2.zip › Figure 1-figure supplement 2. source data 2. Original westerns part A/S2A Puro U343.tif]

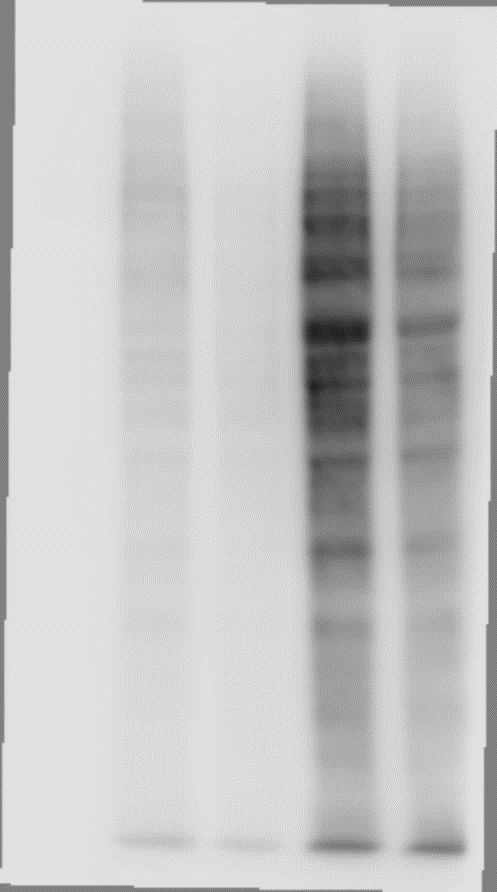

Supplement: Figure 1—figure supplement 2—source data 2. [file elife-98152-fig1-figsupp2-data2.zip › Figure 1-figure supplement 2. source data 2. Original westerns part A/S2A puro.tif]

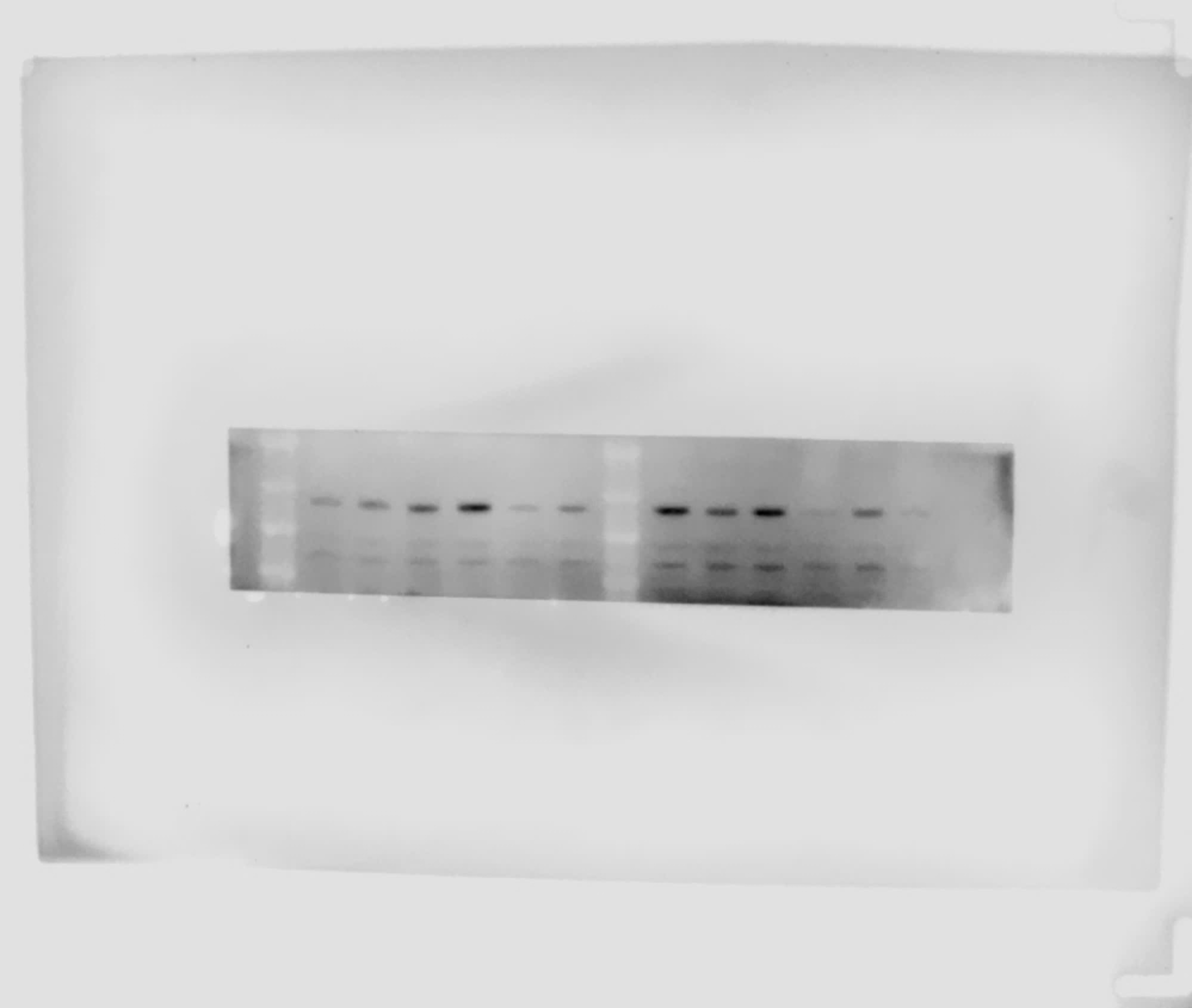

Supplement: Figure 1—figure supplement 2—source data 2. [file elife-98152-fig1-figsupp2-data2.zip › Figure 1-figure supplement 2. source data 2. Original westerns part A/S2A SERBP1.tif]

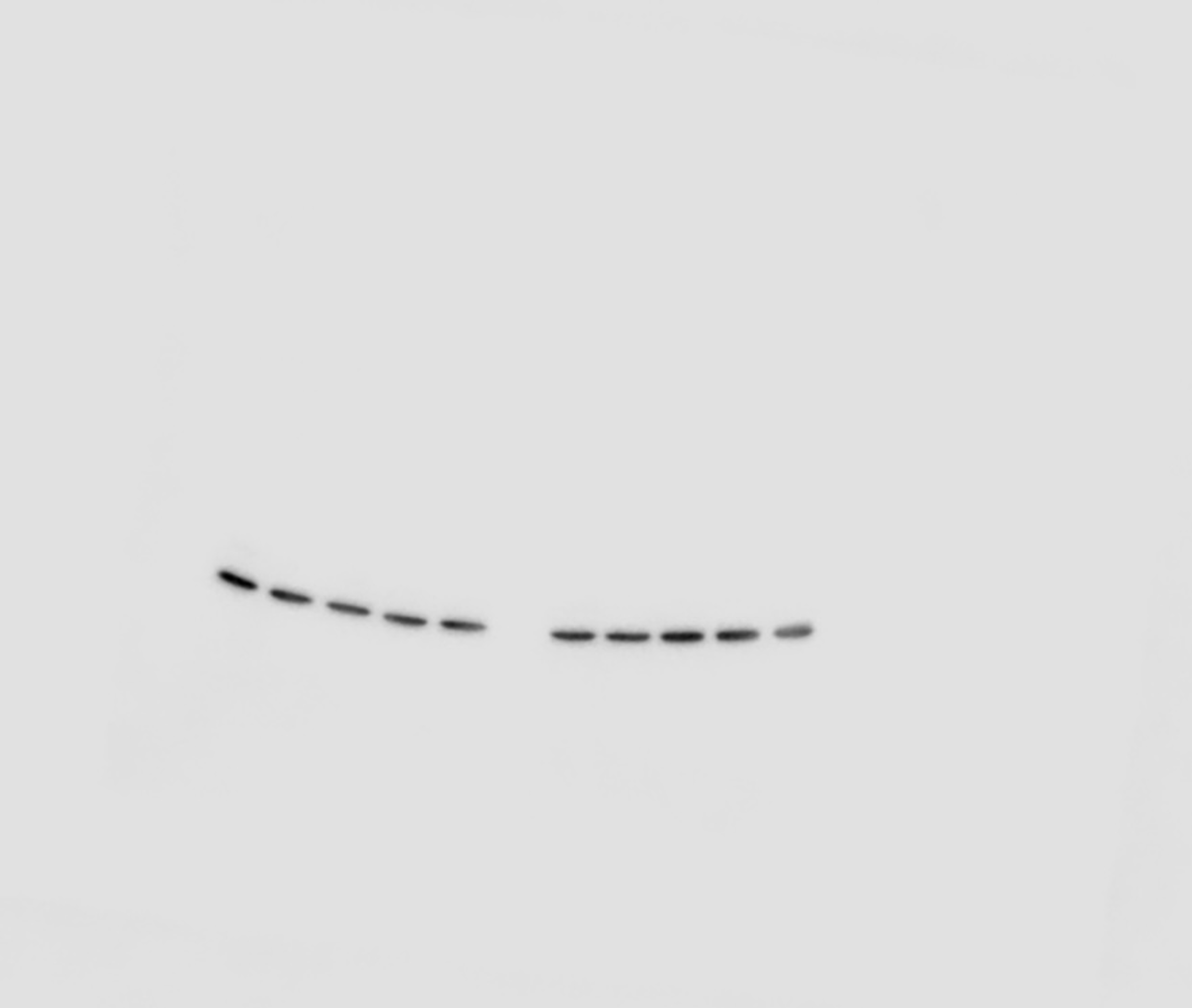

Supplement: Figure 1—figure supplement 2—source data 2. [file elife-98152-fig1-figsupp2-data2.zip › Figure 1-figure supplement 2. source data 2. Original westerns part A/S2A u343 Actin.tif]

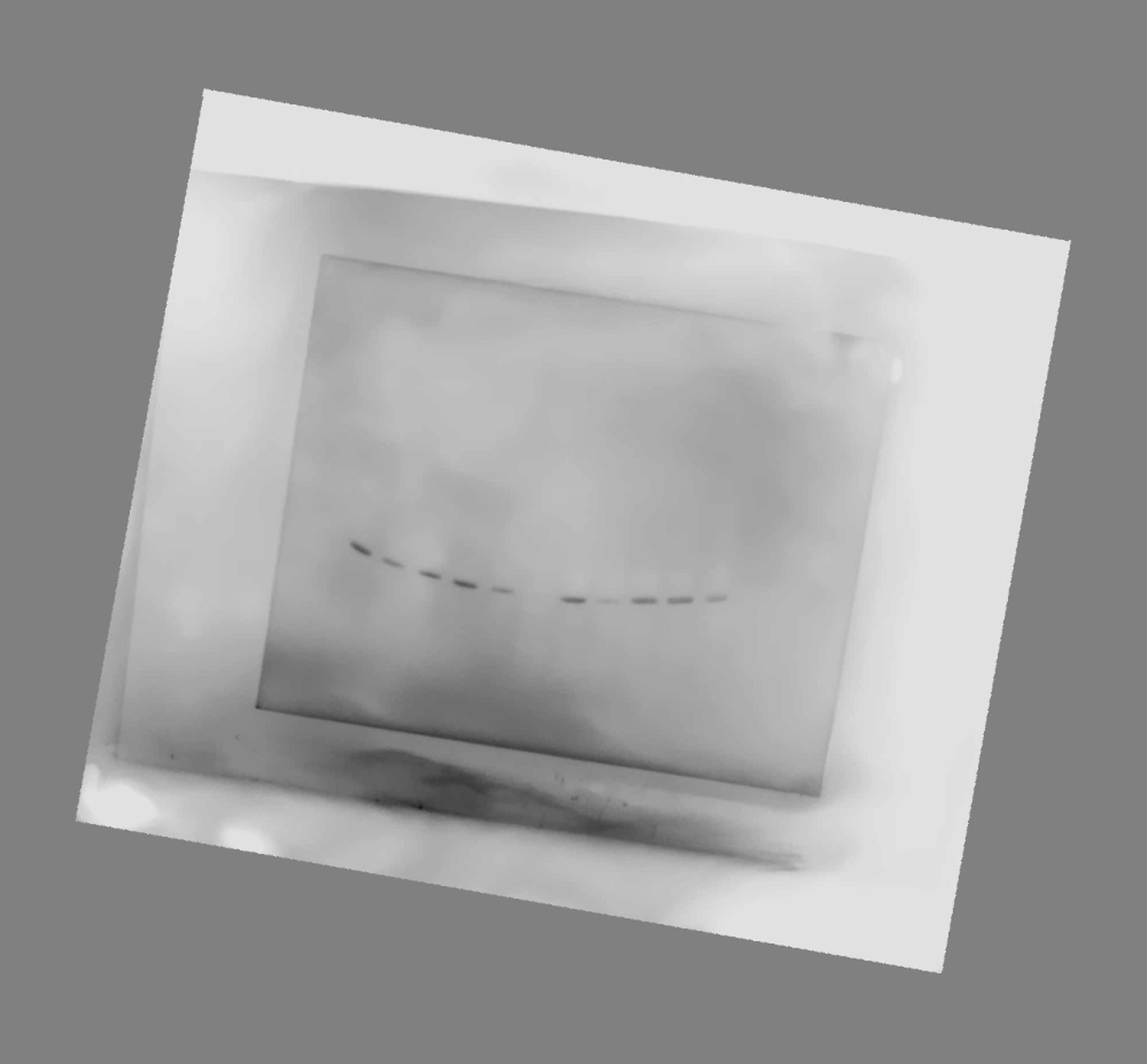

Supplement: Figure 1—figure supplement 2—source data 2. [file elife-98152-fig1-figsupp2-data2.zip › Figure 1-figure supplement 2. source data 2. Original westerns part A/S2A u343 Serbp1.tif]

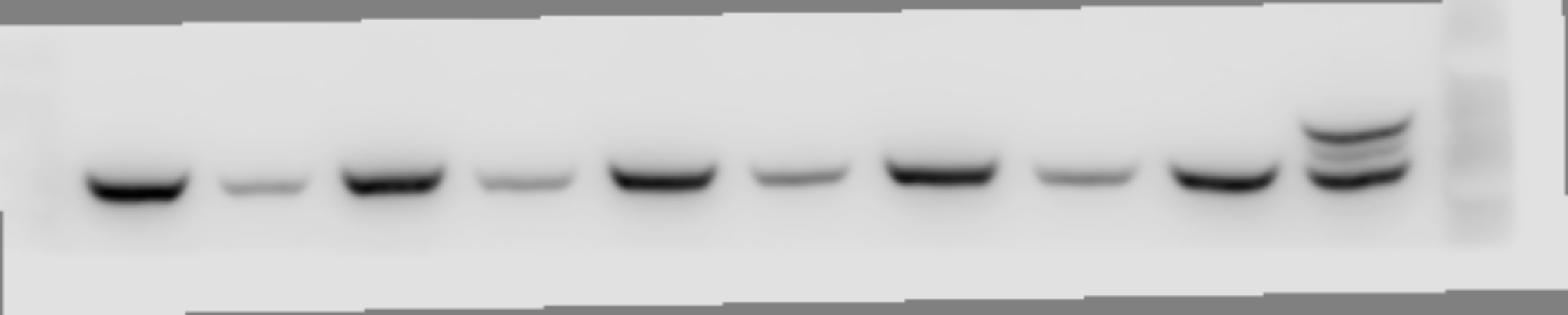

Supplement: Figure 2—source data 2. [file elife-98152-fig2-data2.zip › Figure 2 - source data 2. Original westerns for Figure 2B/2B serbp1 U251.tiff]

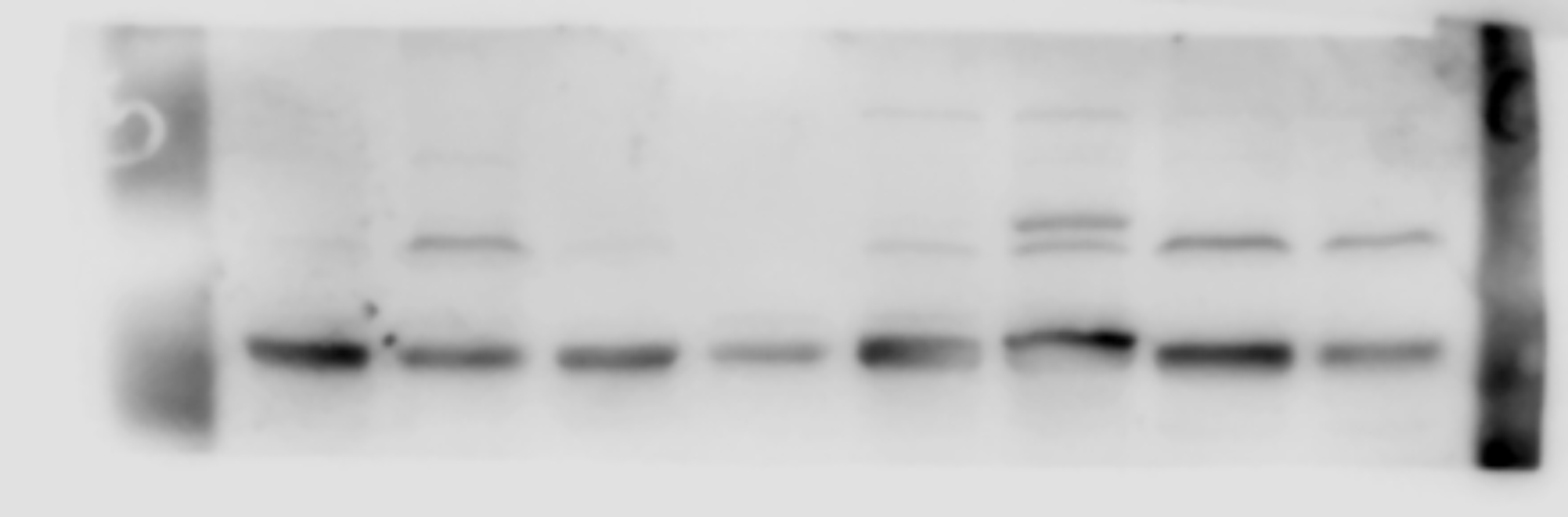

Supplement: Figure 2—source data 2. [file elife-98152-fig2-data2.zip › Figure 2 - source data 2. Original westerns for Figure 2B/2B Serbp1.tiff]

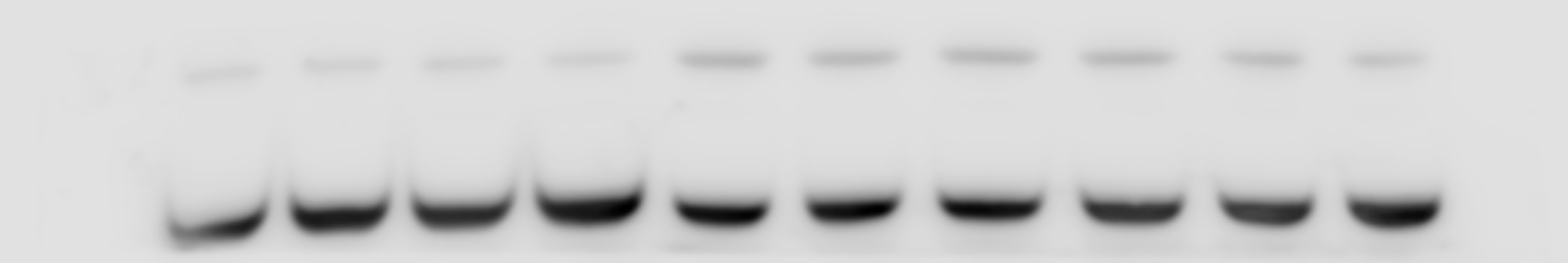

Supplement: Figure 2—source data 2. [file elife-98152-fig2-data2.zip › Figure 2 - source data 2. Original westerns for Figure 2B/2B Tub (2).tiff]

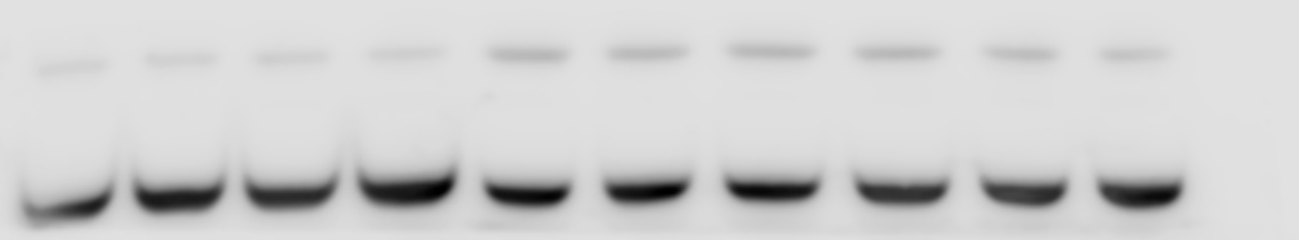

Supplement: Figure 2—source data 2. [file elife-98152-fig2-data2.zip › Figure 2 - source data 2. Original westerns for Figure 2B/2B Tub.tif]

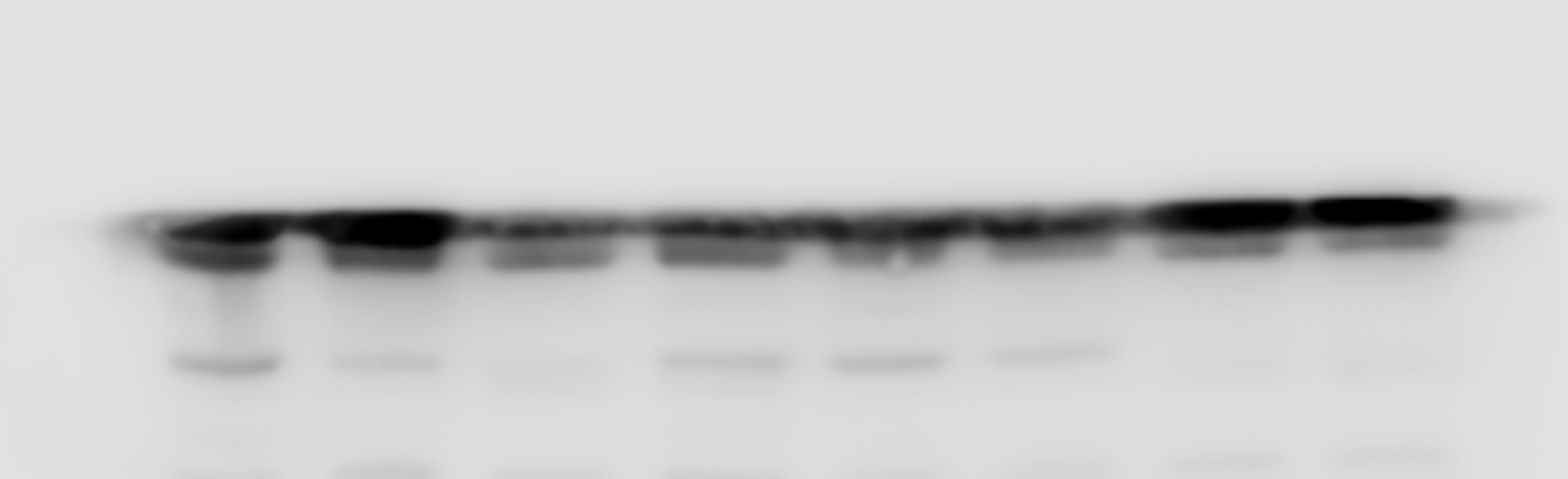

Supplement: Figure 2—source data 2. [file elife-98152-fig2-data2.zip › Figure 2 - source data 2. Original westerns for Figure 2B/2B Tub.tiff]

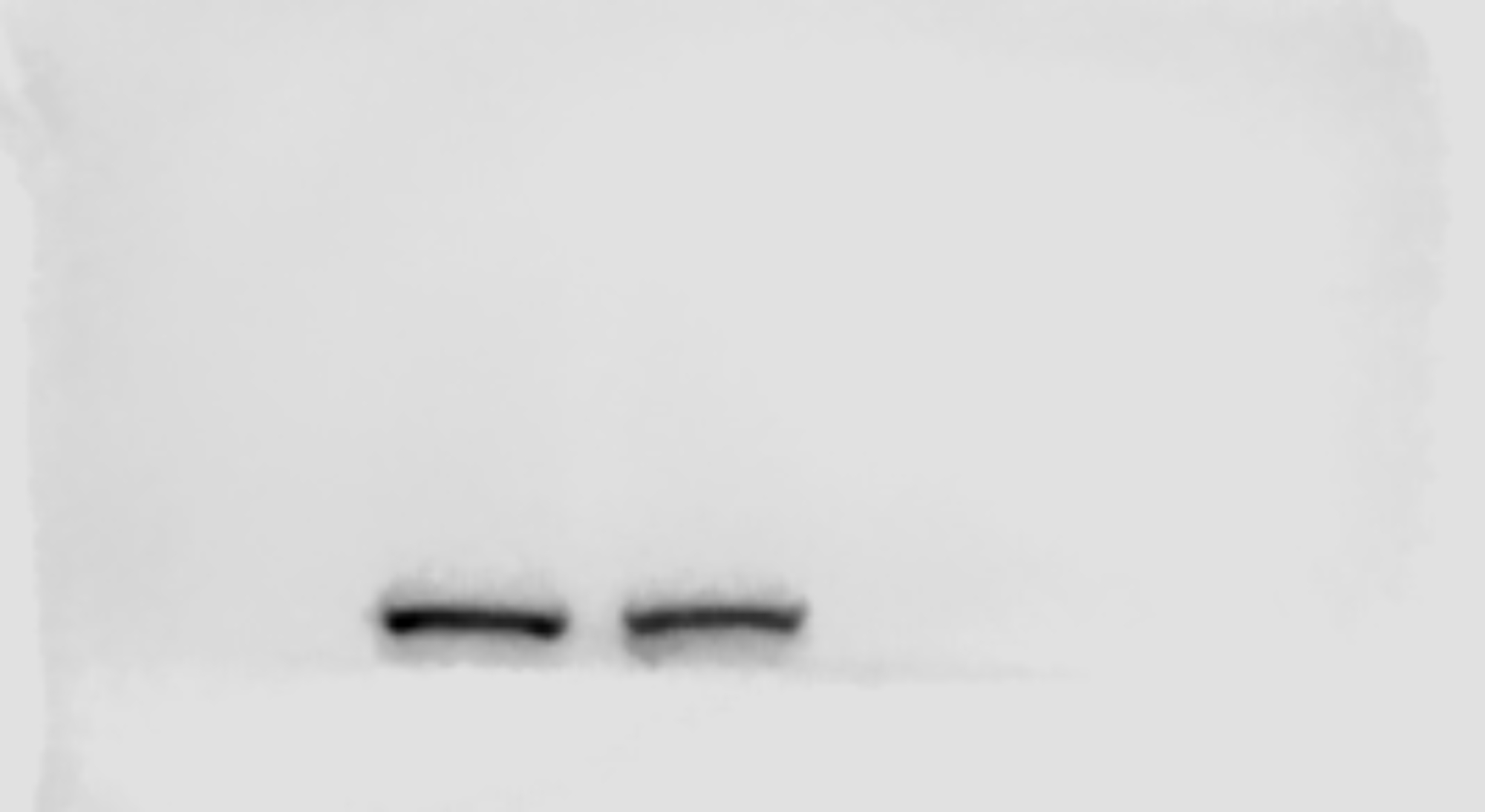

Supplement: Figure 4—source data 7. [file elife-98152-fig4-data7.zip › Figure 4- source data 7. Original westerns in Figure 4/4A GAPDH.tiff]

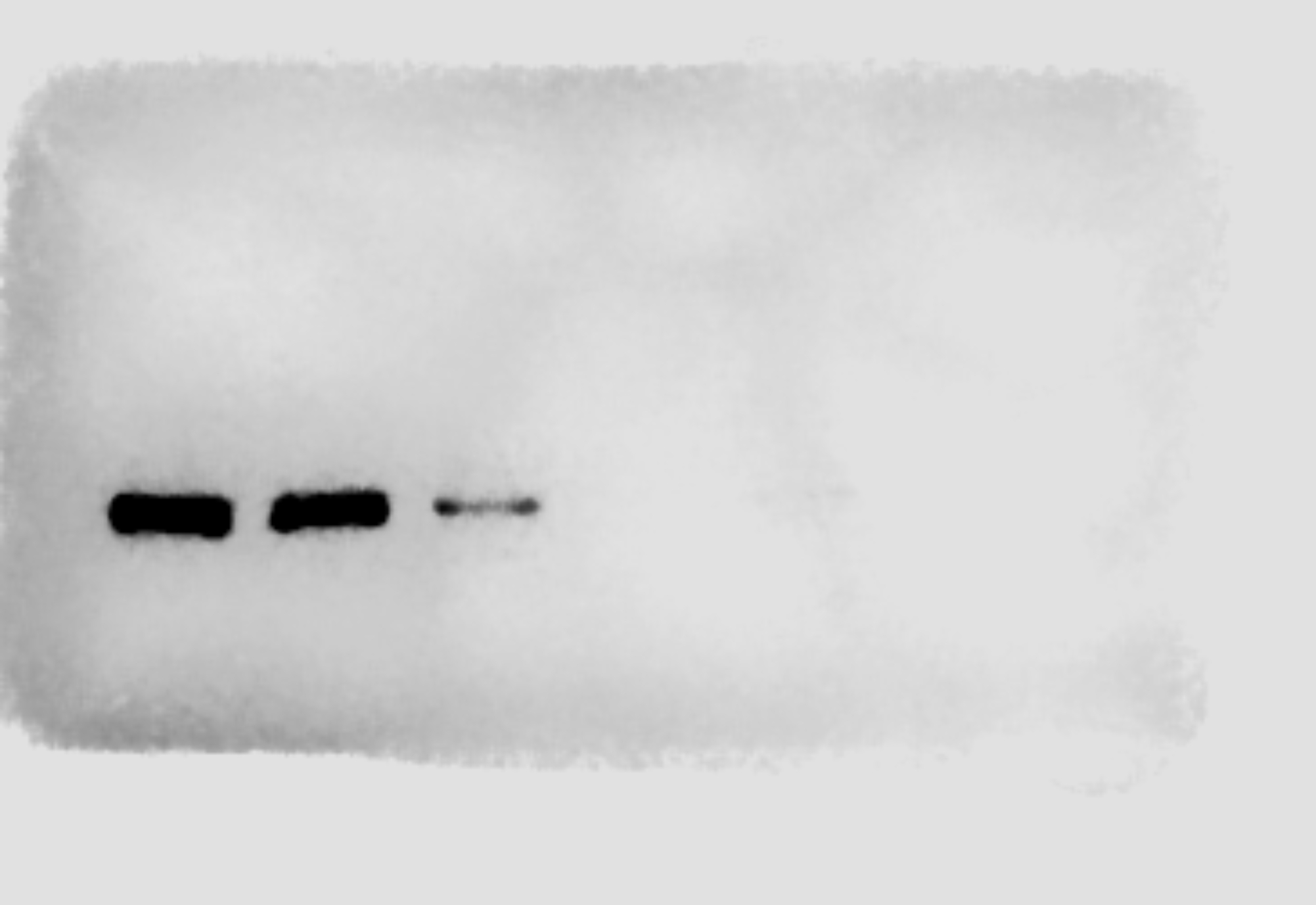

Supplement: Figure 4—source data 7. [file elife-98152-fig4-data7.zip › Figure 4- source data 7. Original westerns in Figure 4/4A parp.tiff]

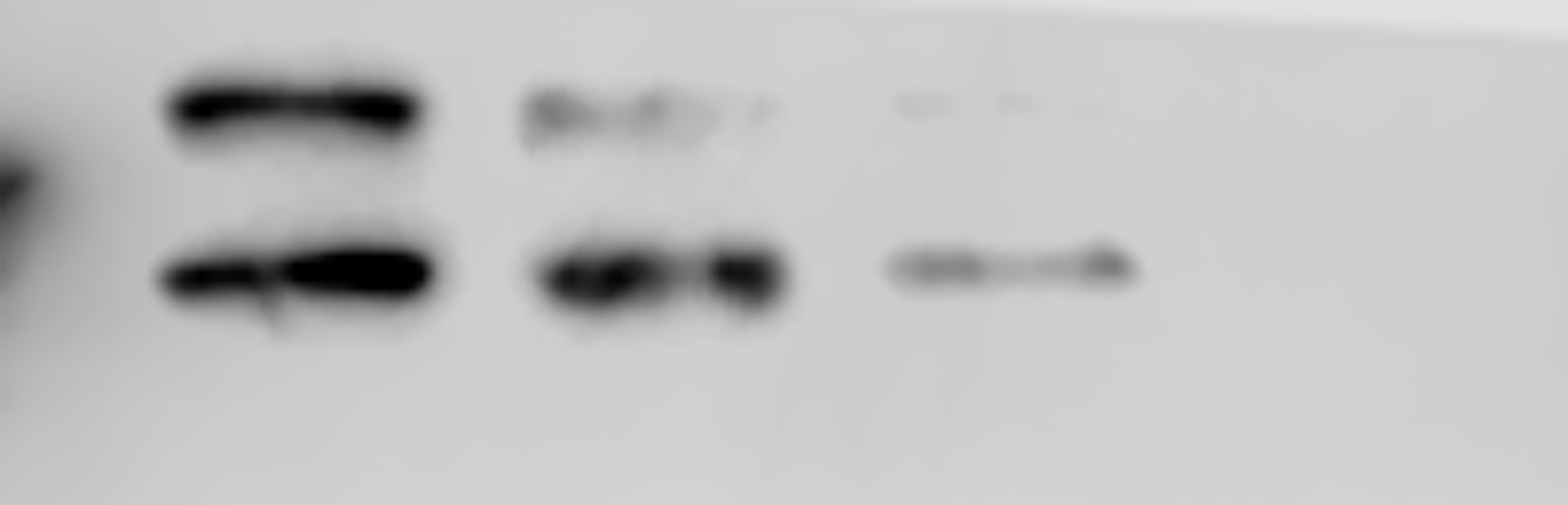

Supplement: Figure 4—source data 7. [file elife-98152-fig4-data7.zip › Figure 4- source data 7. Original westerns in Figure 4/4A syn.tiff]

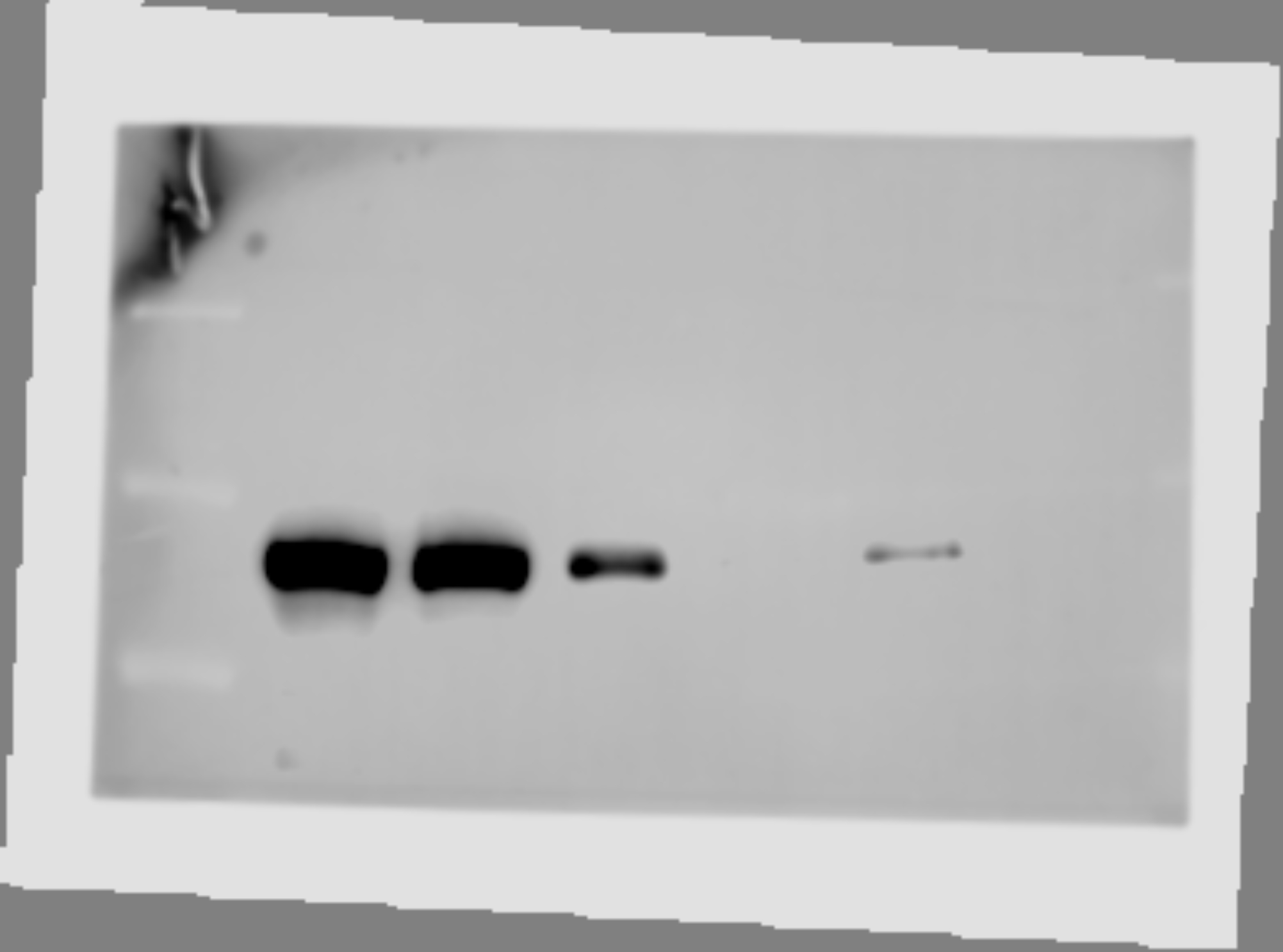

Supplement: Figure 4—source data 7. [file elife-98152-fig4-data7.zip › Figure 4- source data 7. Original westerns in Figure 4/4ANCL.tiff]

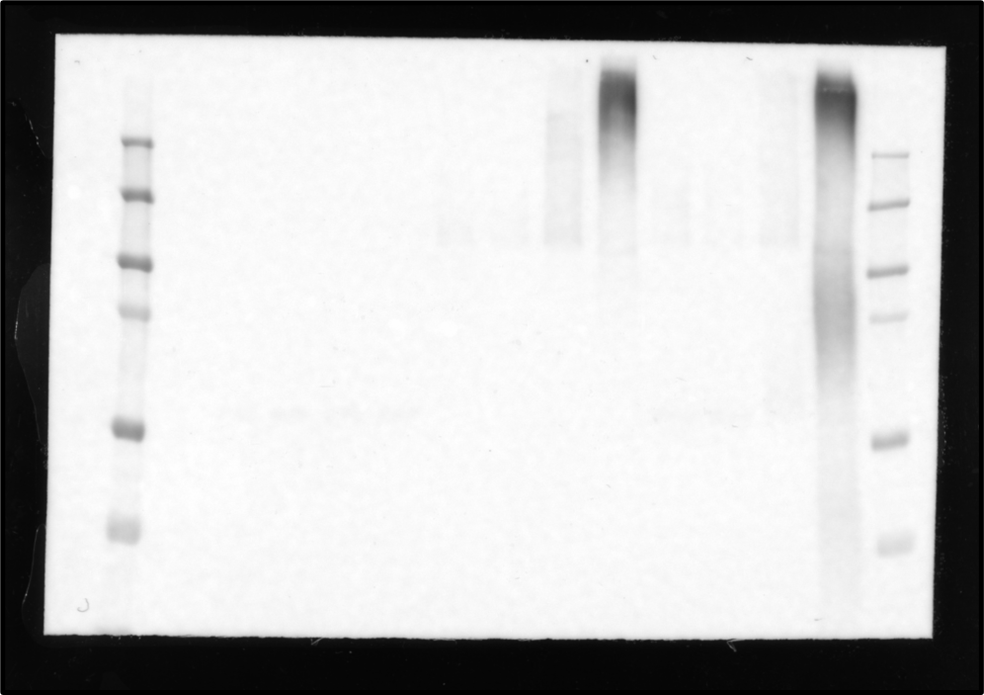

Supplement: Figure 4—source data 7. [file elife-98152-fig4-data7.zip › Figure 4- source data 7. Original westerns in Figure 4/4E par.tif]

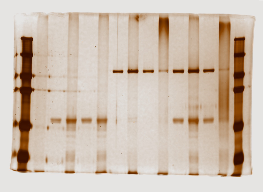

Supplement: Figure 4—source data 7. [file elife-98152-fig4-data7.zip › Figure 4- source data 7. Original westerns in Figure 4/4E staining.tif]

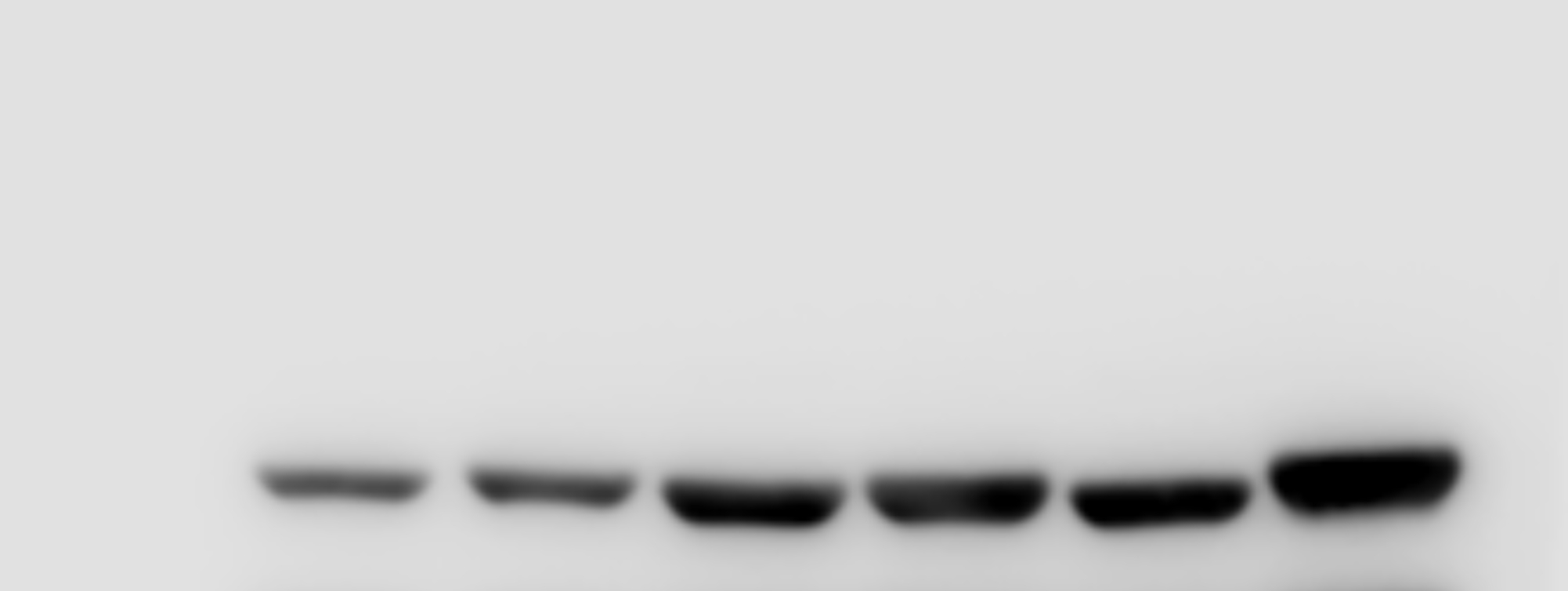

Supplement: Figure 4—source data 7. [file elife-98152-fig4-data7.zip › Figure 4- source data 7. Original westerns in Figure 4/4G actin.tiff]

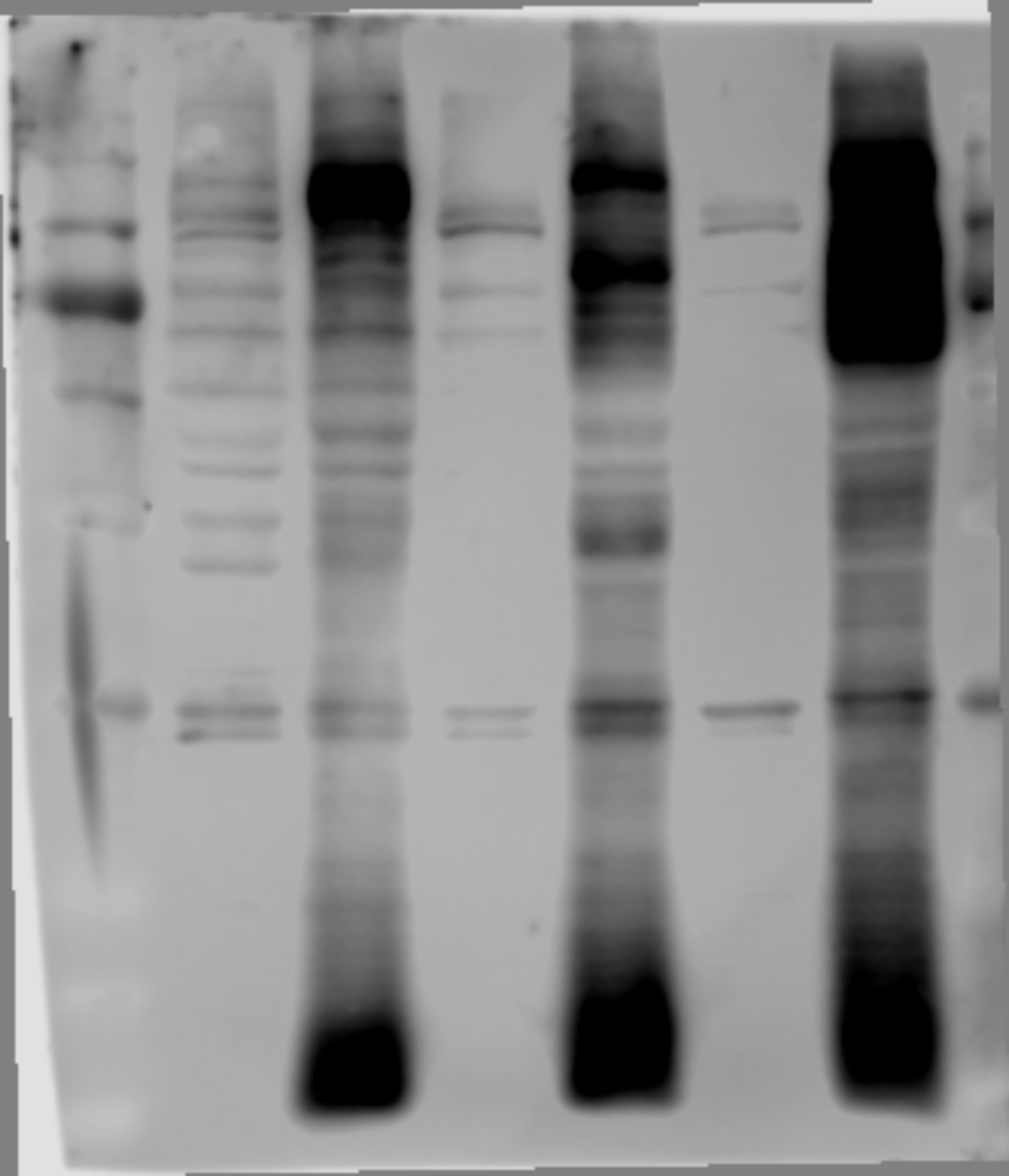

Supplement: Figure 4—source data 7. [file elife-98152-fig4-data7.zip › Figure 4- source data 7. Original westerns in Figure 4/4G par.tiff]

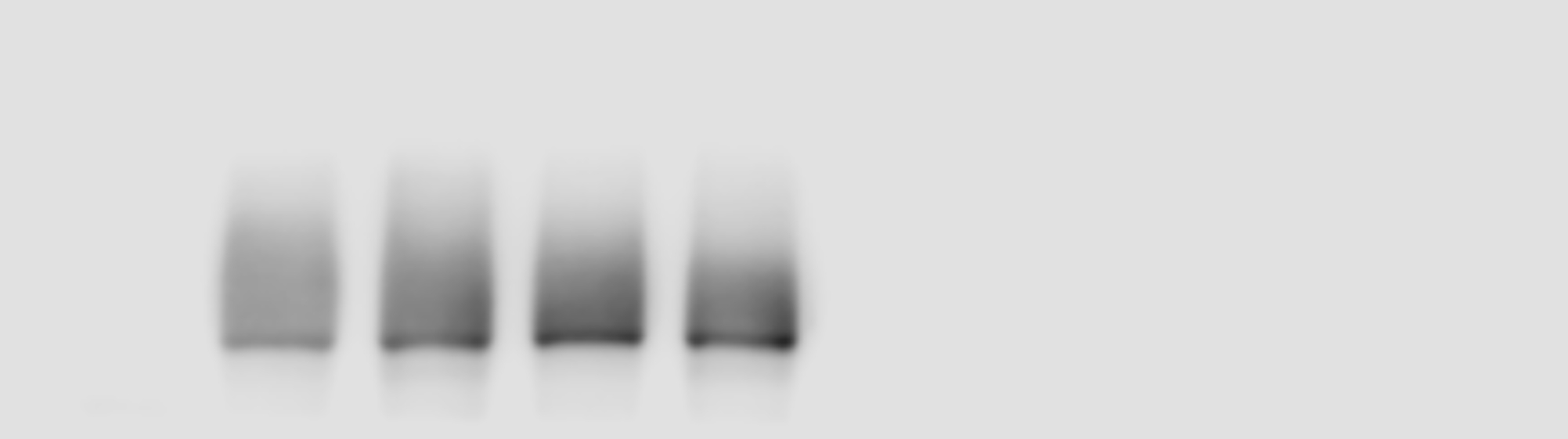

Supplement: Figure 4—source data 7. [file elife-98152-fig4-data7.zip › Figure 4- source data 7. Original westerns in Figure 4/4H INP Flag.tiff]

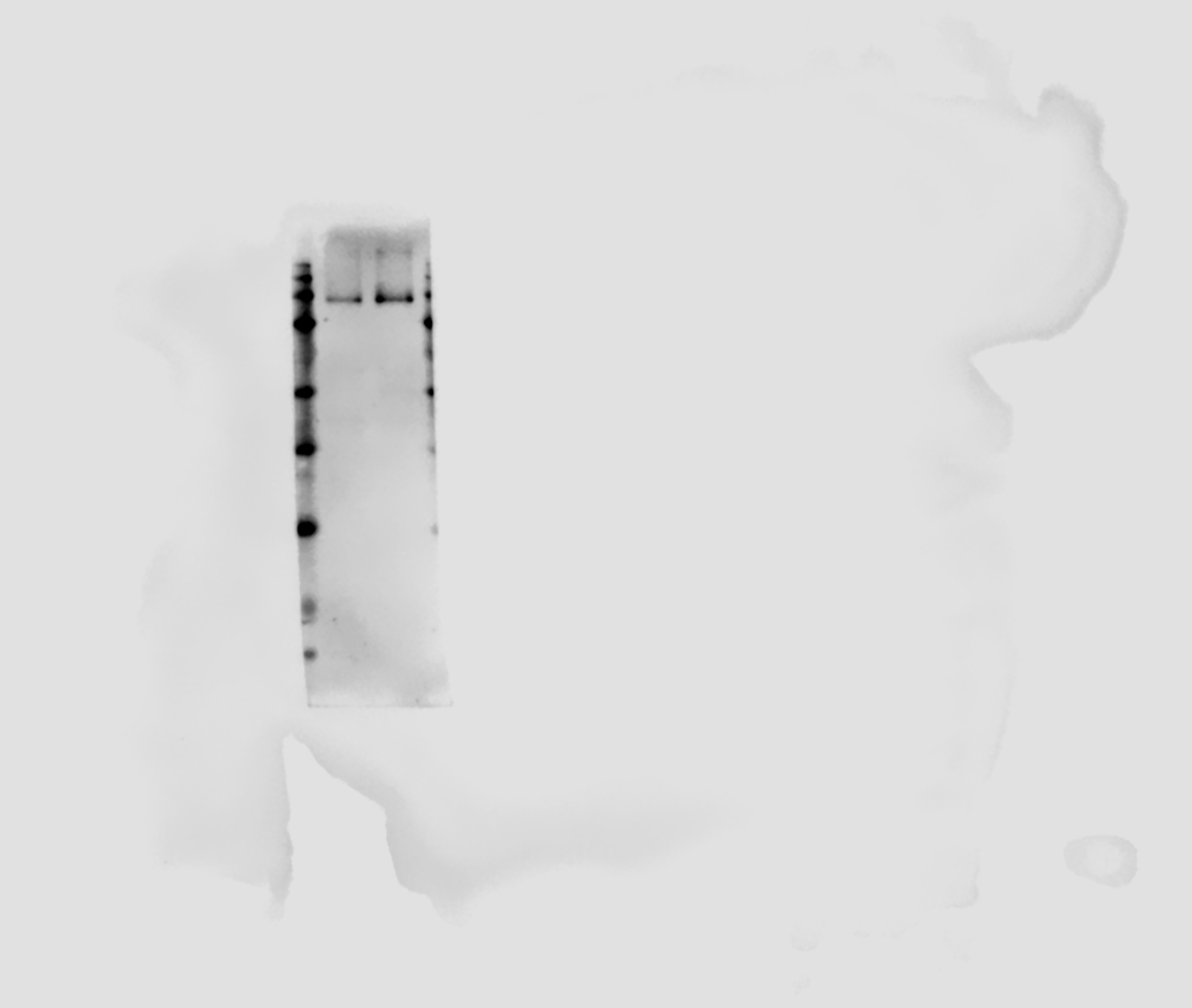

Supplement: Figure 4—source data 7. [file elife-98152-fig4-data7.zip › Figure 4- source data 7. Original westerns in Figure 4/4H IP Flag.tiff]

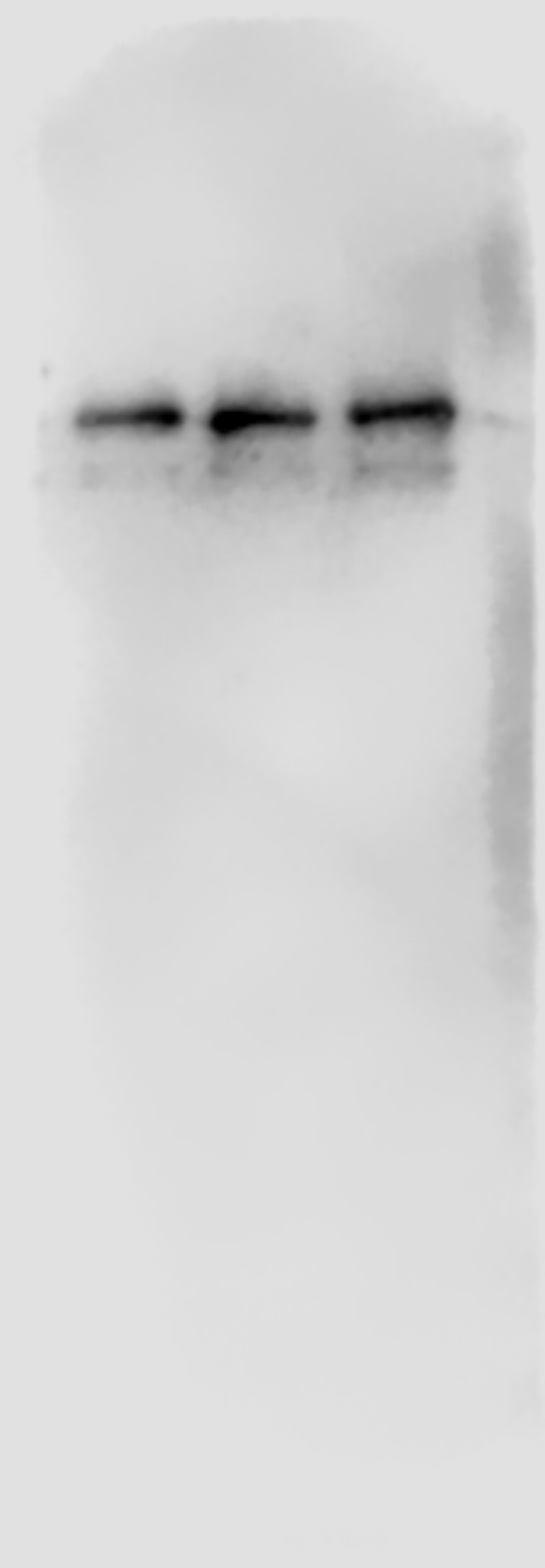

Supplement: Figure 4—source data 7. [file elife-98152-fig4-data7.zip › Figure 4- source data 7. Original westerns in Figure 4/4H IP His.tiff]

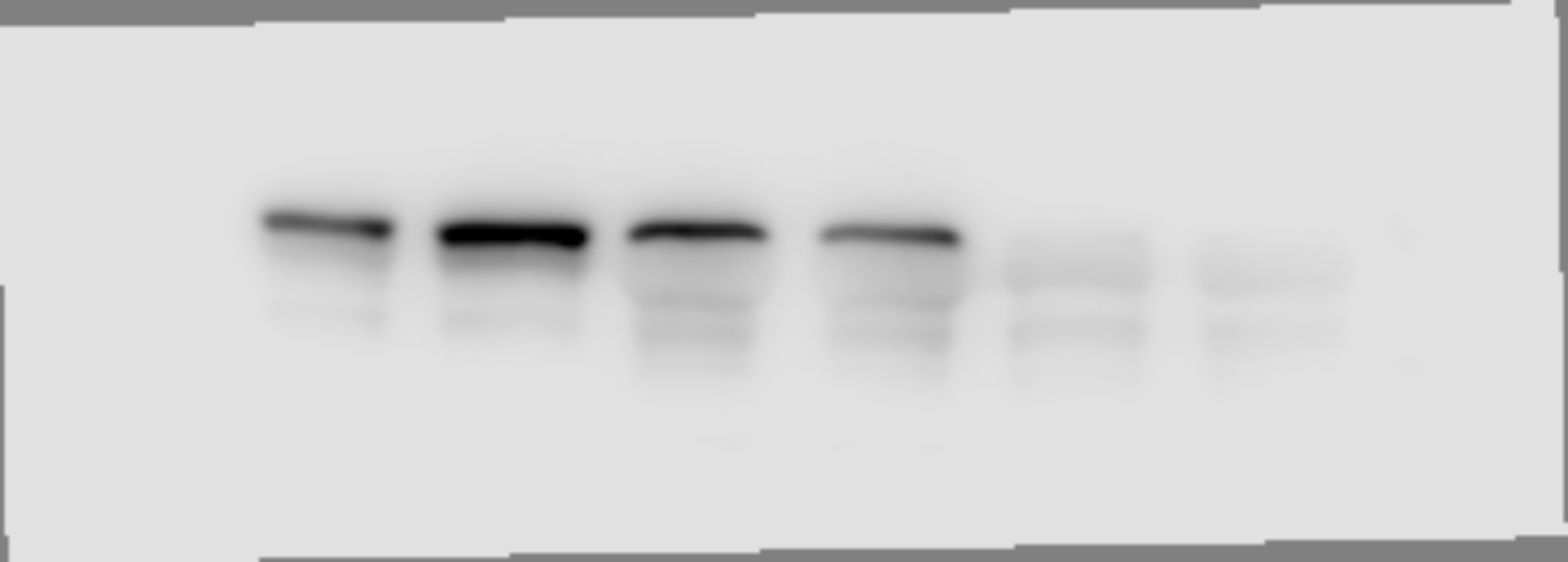

Supplement: Figure 4—source data 7. [file elife-98152-fig4-data7.zip › Figure 4- source data 7. Original westerns in Figure 4/4I G3bp1I.tiff]

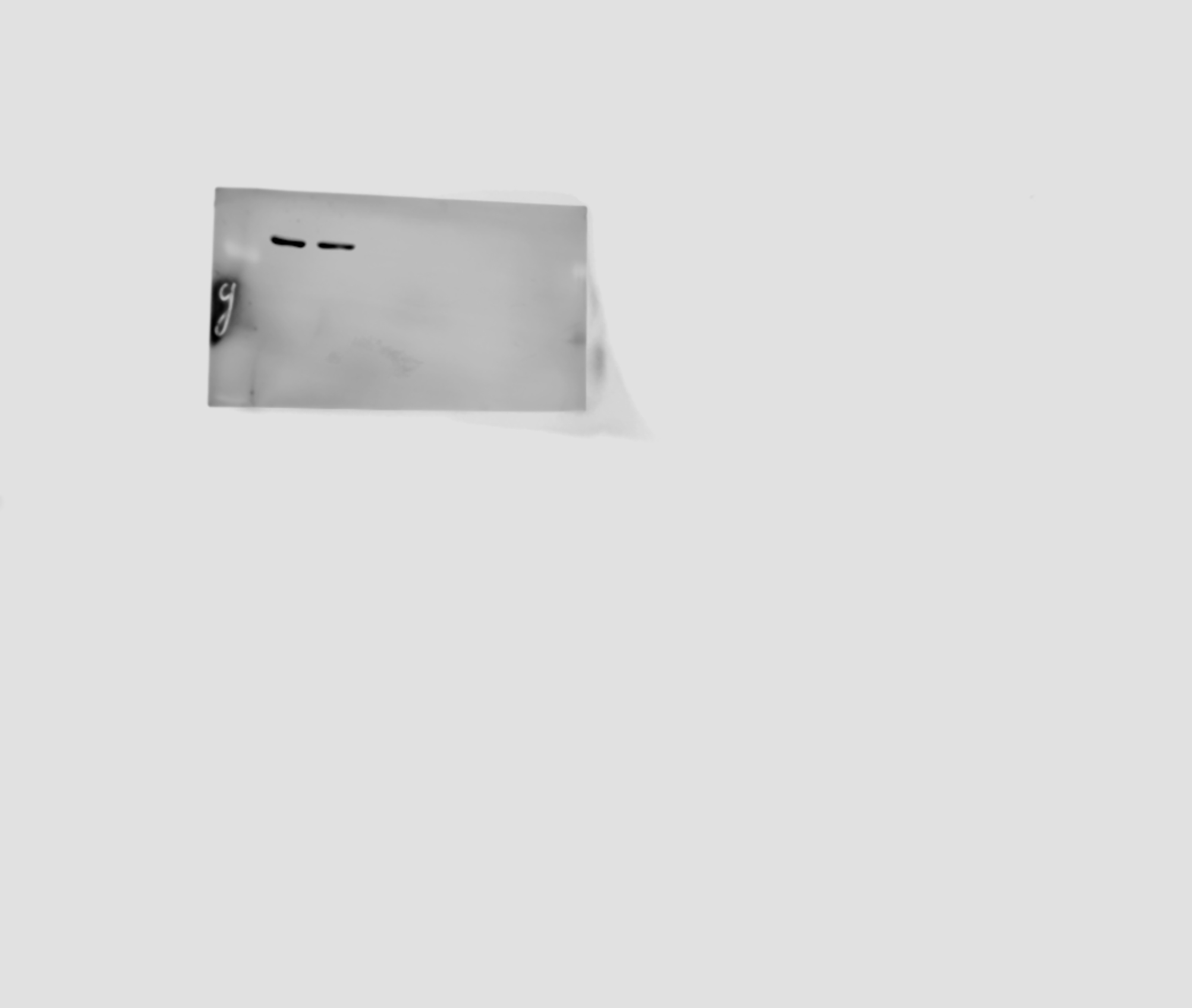

Supplement: Figure 4—source data 7. [file elife-98152-fig4-data7.zip › Figure 4- source data 7. Original westerns in Figure 4/4I gapdh.tiff]

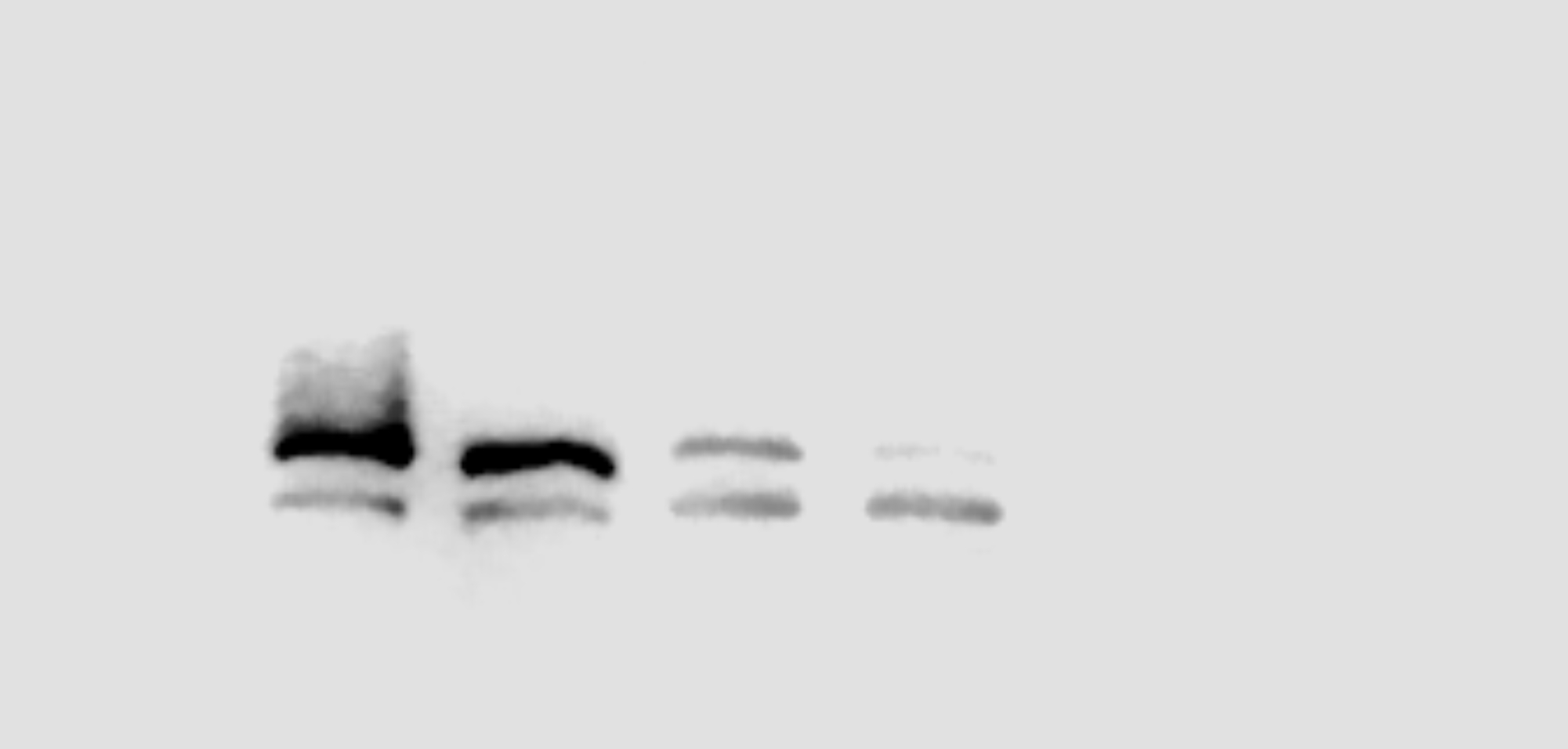

Supplement: Figure 4—source data 7. [file elife-98152-fig4-data7.zip › Figure 4- source data 7. Original westerns in Figure 4/4I parp.tiff]

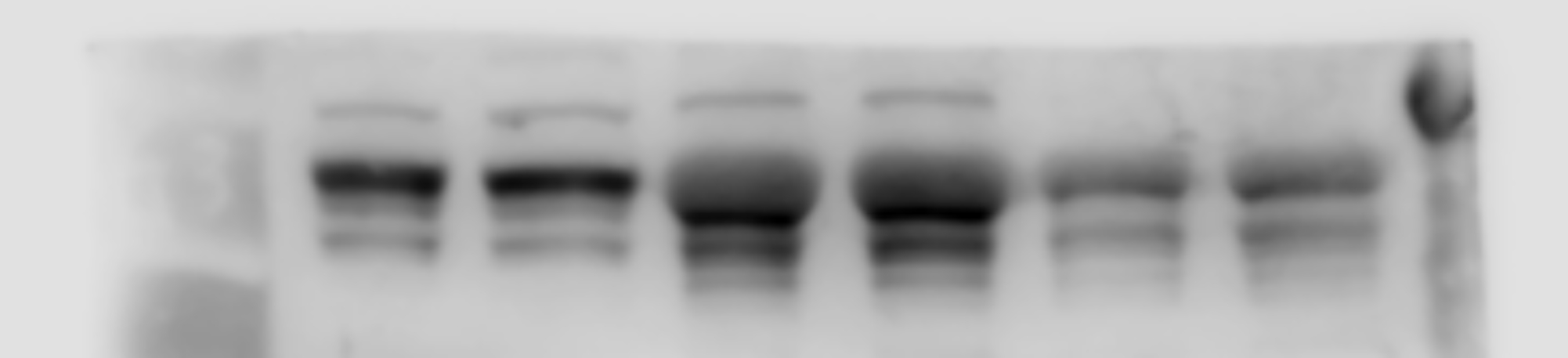

Supplement: Figure 4—source data 7. [file elife-98152-fig4-data7.zip › Figure 4- source data 7. Original westerns in Figure 4/4I serbp.tiff]

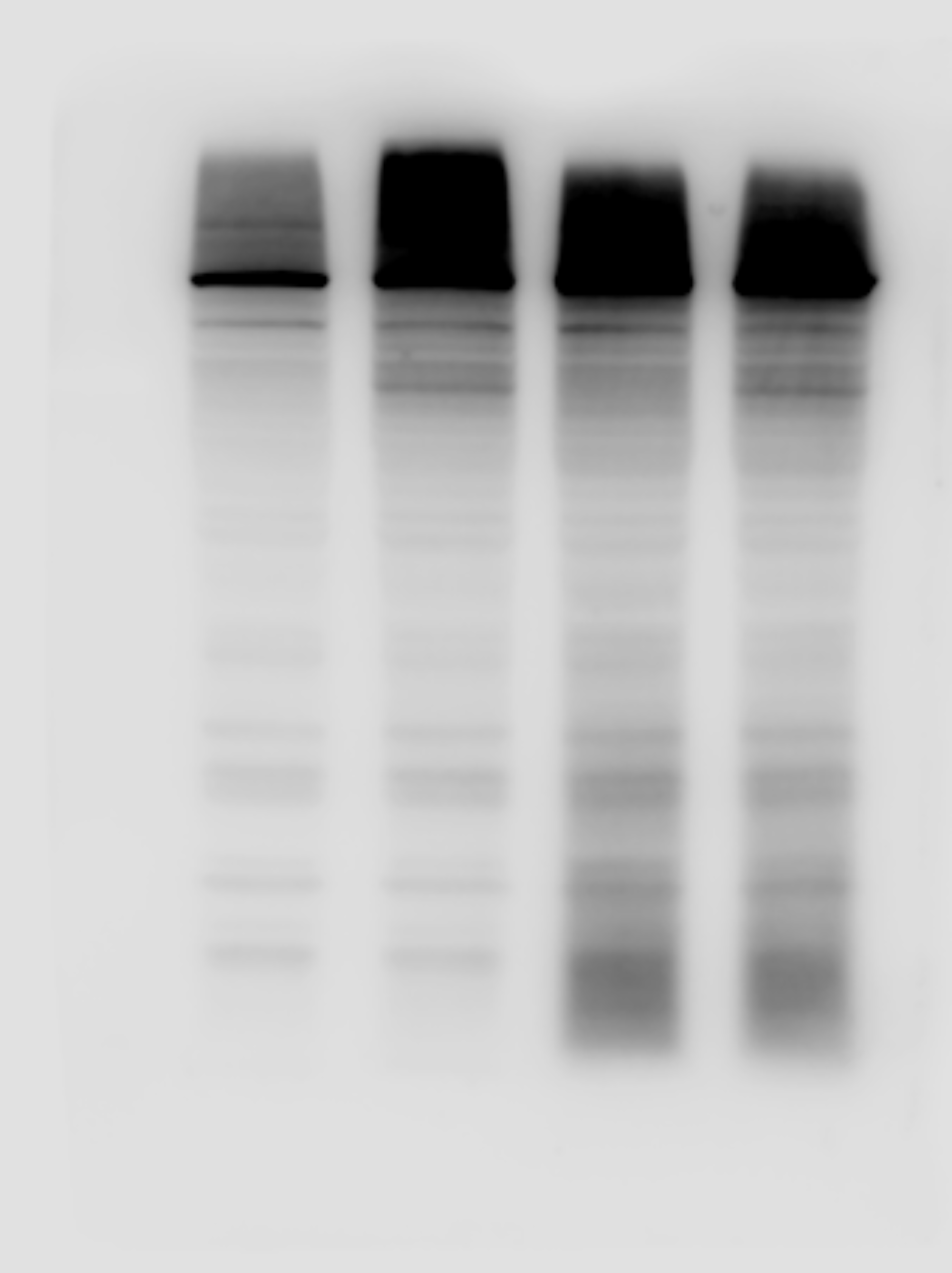

Supplement: Figure 4—source data 7. [file elife-98152-fig4-data7.zip › Figure 4- source data 7. Original westerns in Figure 4/4J par .tiff]

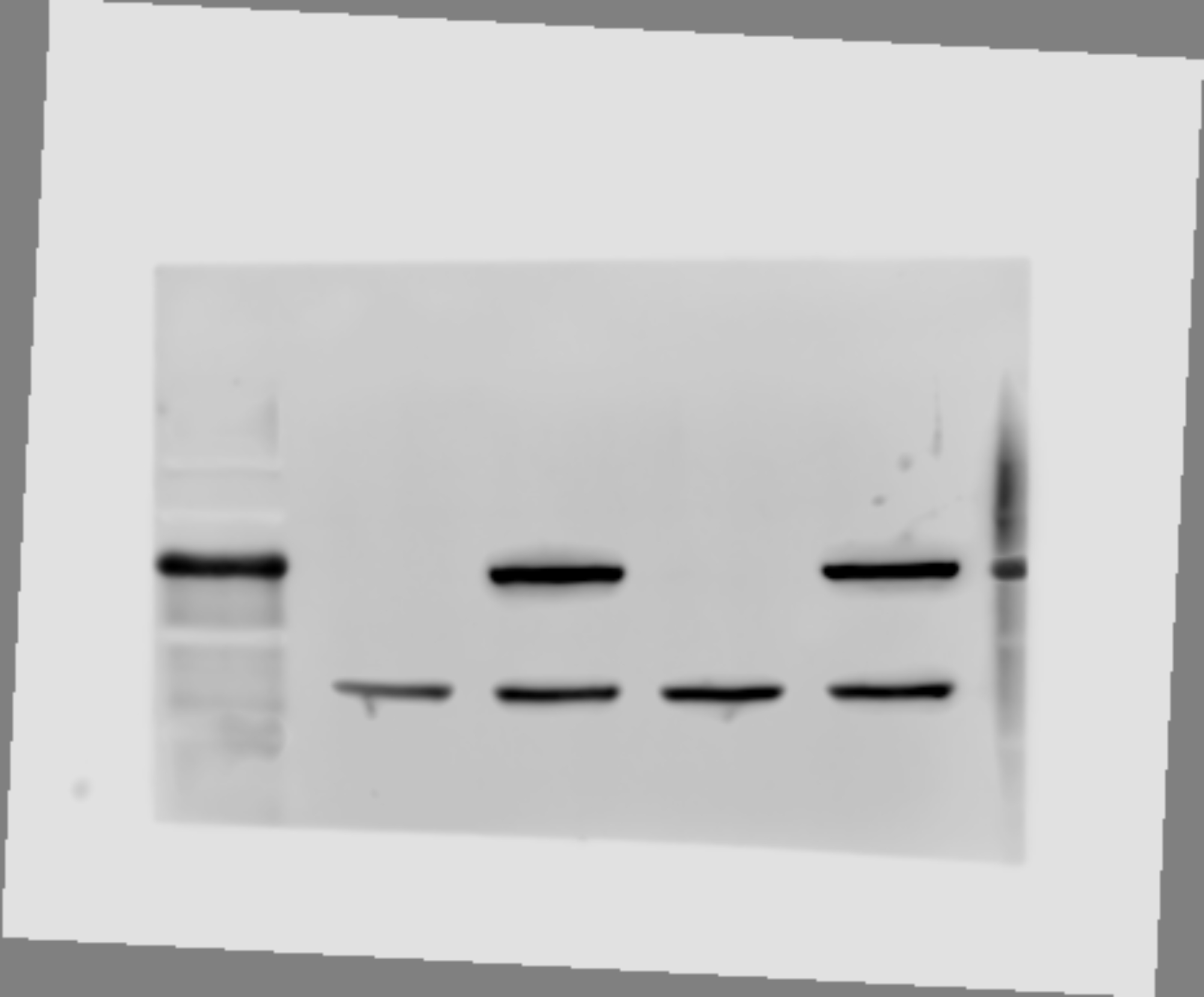

Supplement: Figure 4—source data 7. [file elife-98152-fig4-data7.zip › Figure 4- source data 7. Original westerns in Figure 4/4J serbp1.tiff]

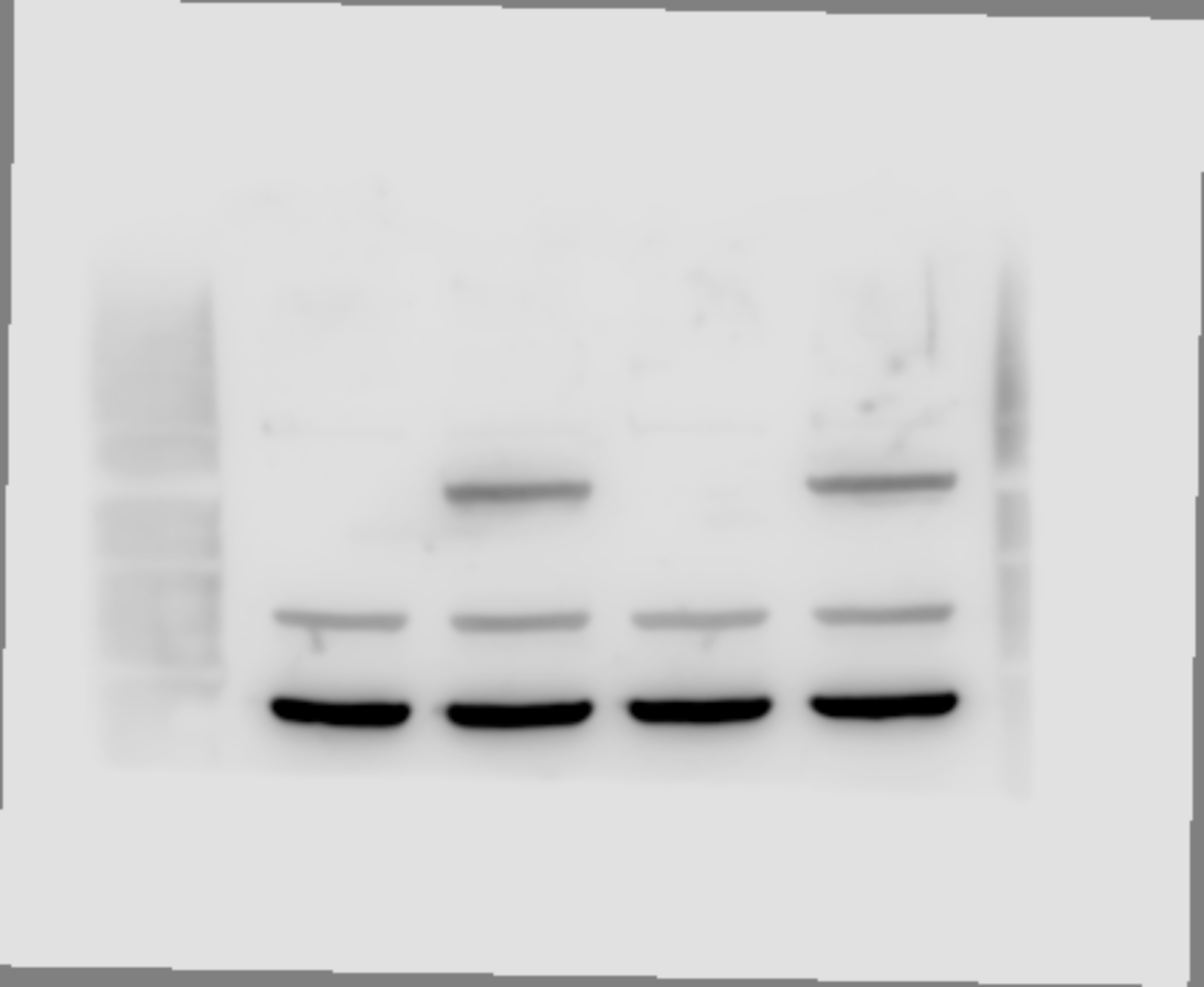

Supplement: Figure 4—source data 7. [file elife-98152-fig4-data7.zip › Figure 4- source data 7. Original westerns in Figure 4/4J tub.tiff]

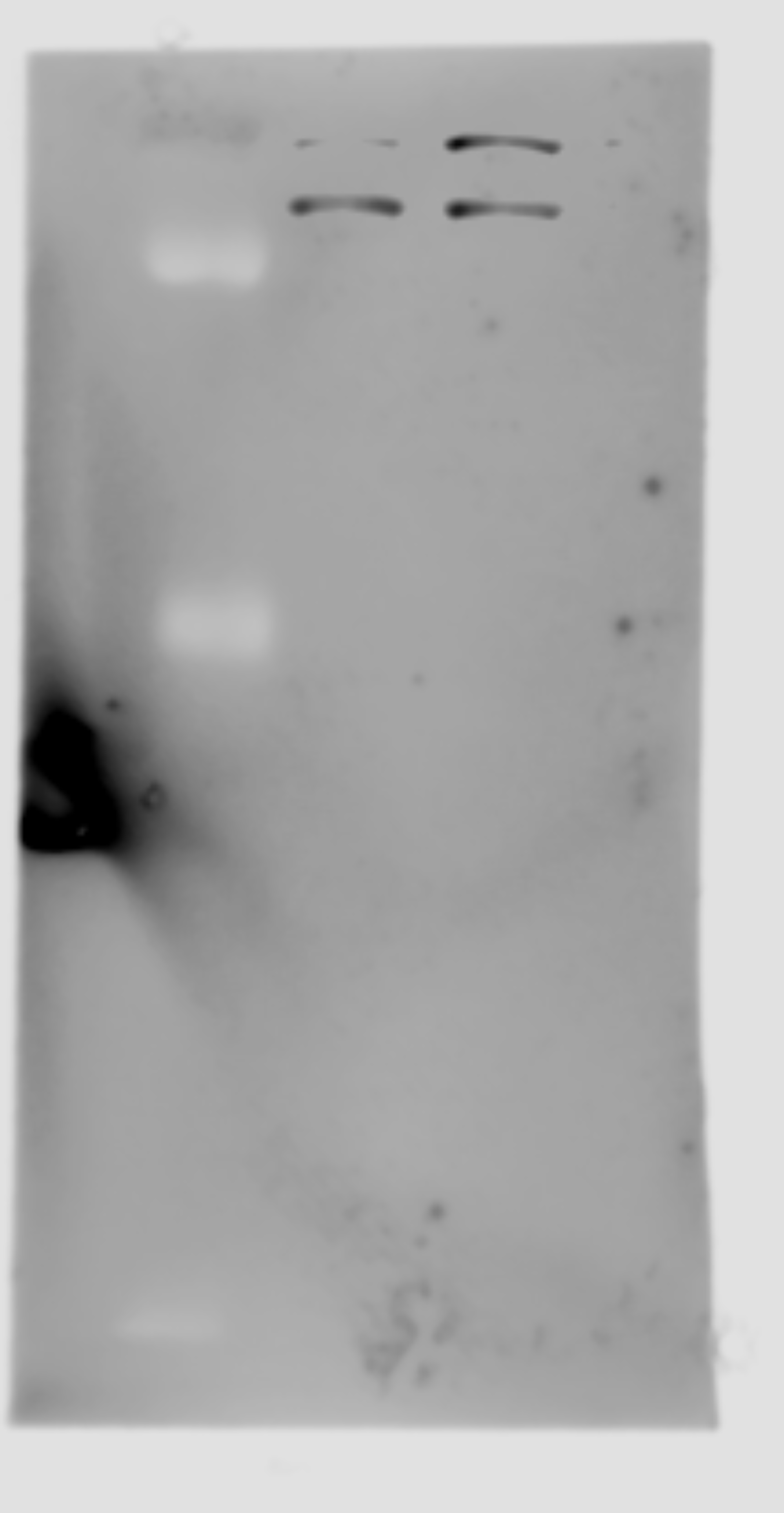

Supplement: Figure 4—source data 7. [file elife-98152-fig4-data7.zip › Figure 4- source data 7. Original westerns in Figure 4/INp His 4H.tif]

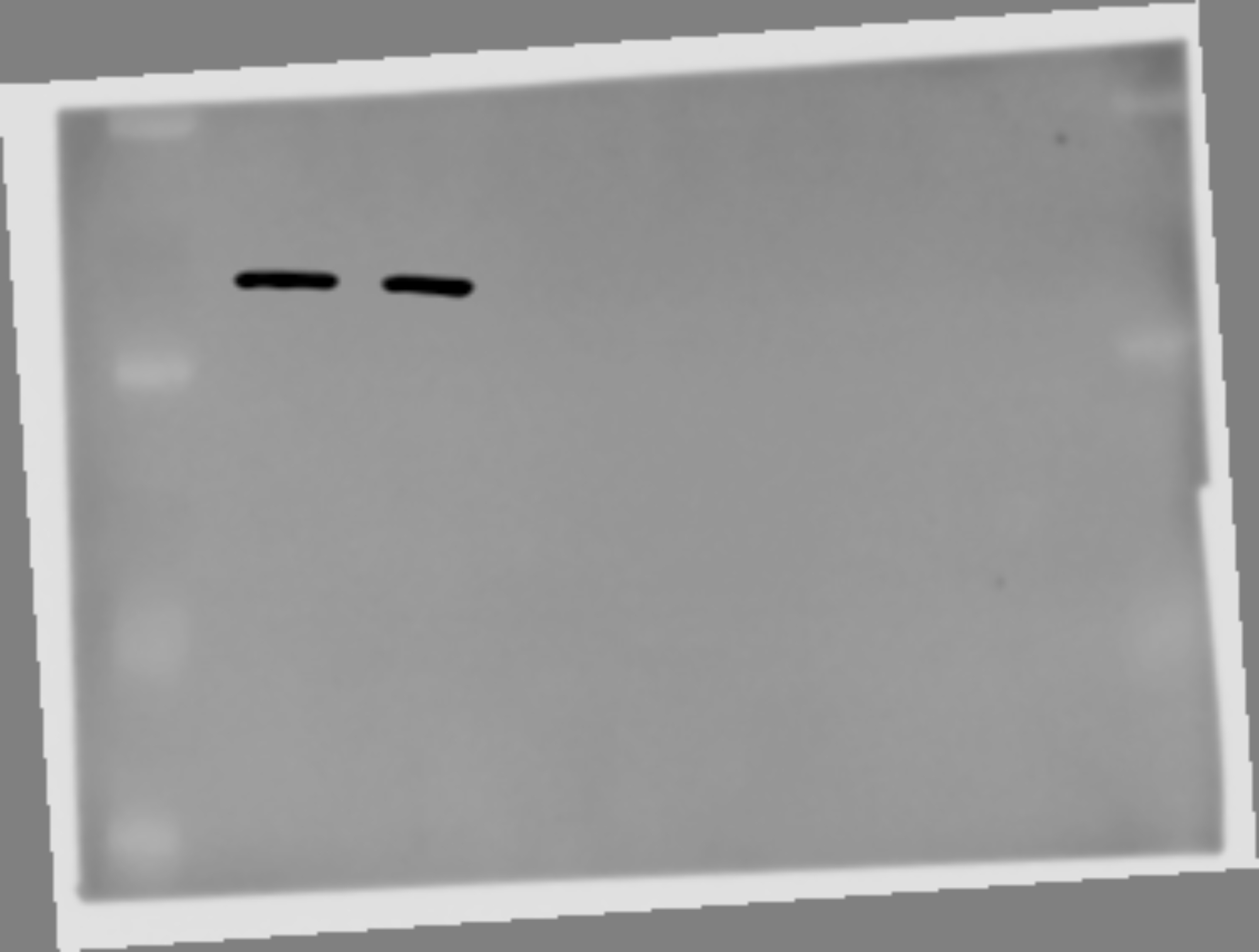

Supplement: Figure 4—figure supplement 1—source data 2. [file elife-98152-fig4-figsupp1-data2.zip › Figure 4-figure supplement 1. source data 2. Original westerns part B/Sup 4B gapdh.tiff]

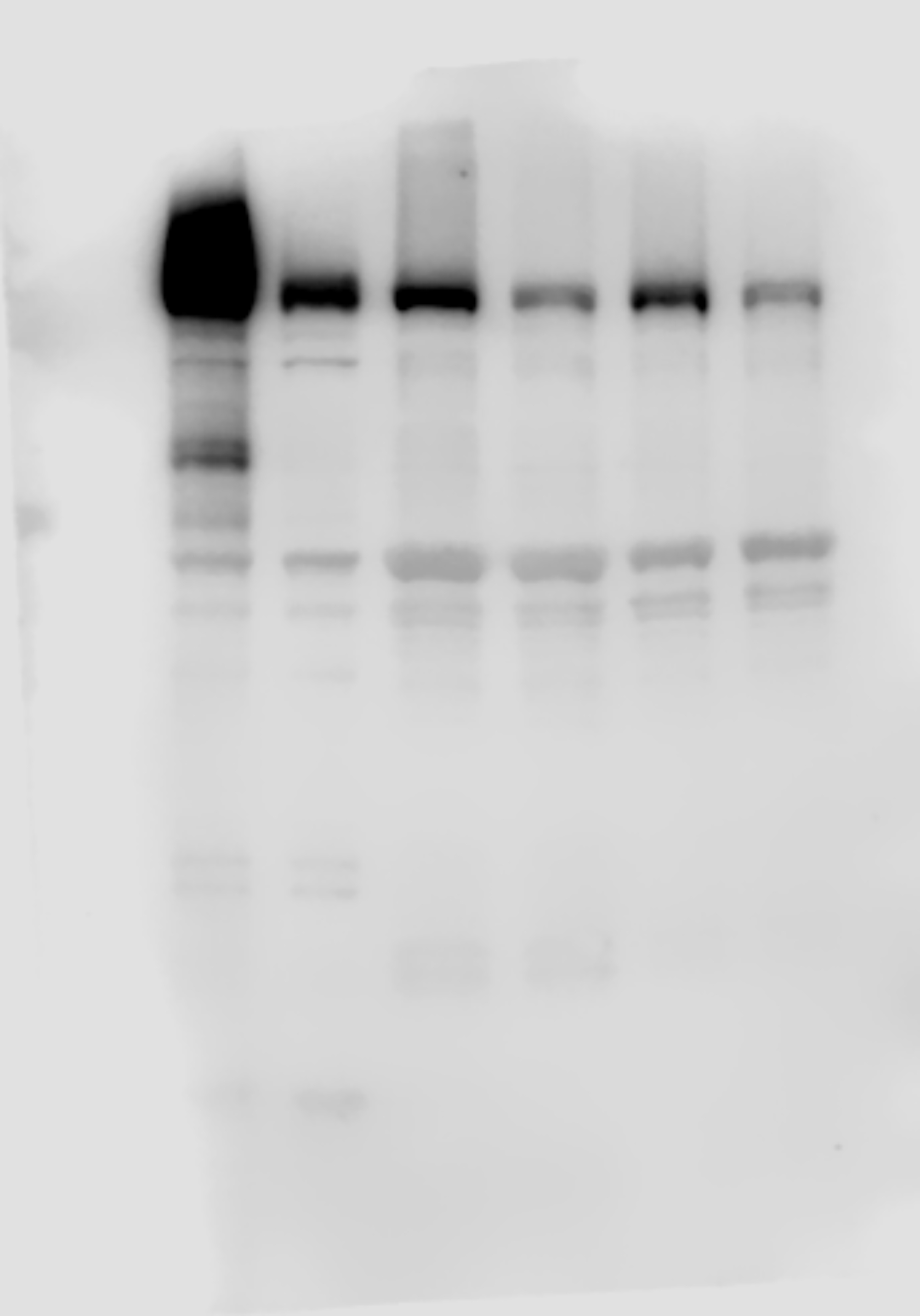

Supplement: Figure 4—figure supplement 1—source data 2. [file elife-98152-fig4-figsupp1-data2.zip › Figure 4-figure supplement 1. source data 2. Original westerns part B/Sup 4B par.tiff]

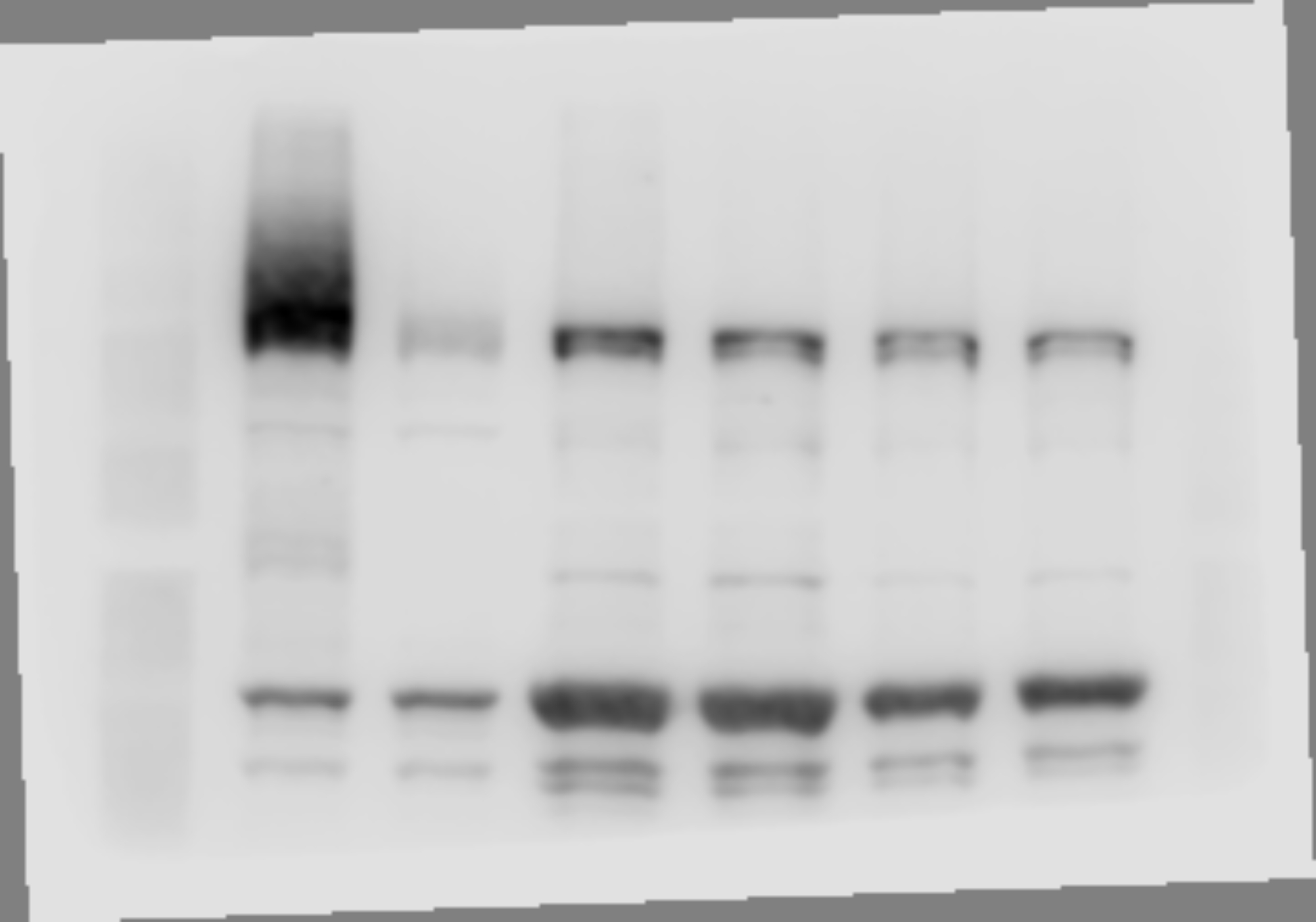

Supplement: Figure 4—figure supplement 1—source data 2. [file elife-98152-fig4-figsupp1-data2.zip › Figure 4-figure supplement 1. source data 2. Original westerns part B/Sup4B serbp1.tiff]

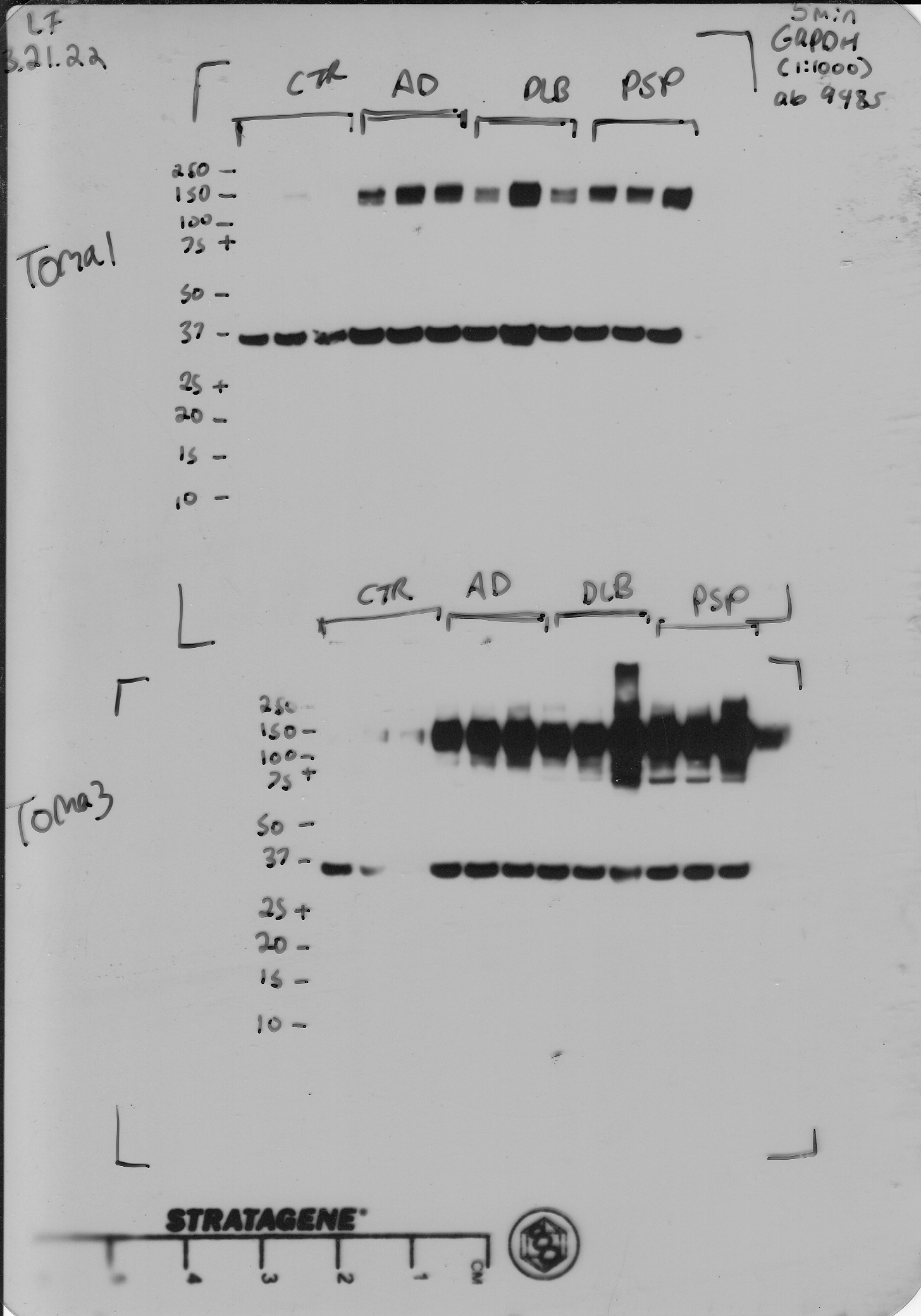

Supplement: Figure 7—source data 2. [file elife-98152-fig7-data2.zip › Figure 7- source data 2. Original westerns in Figure 7D/#2 3.21.22 GAPDH TOMA1.TOMA3 5 min_0001.tif]

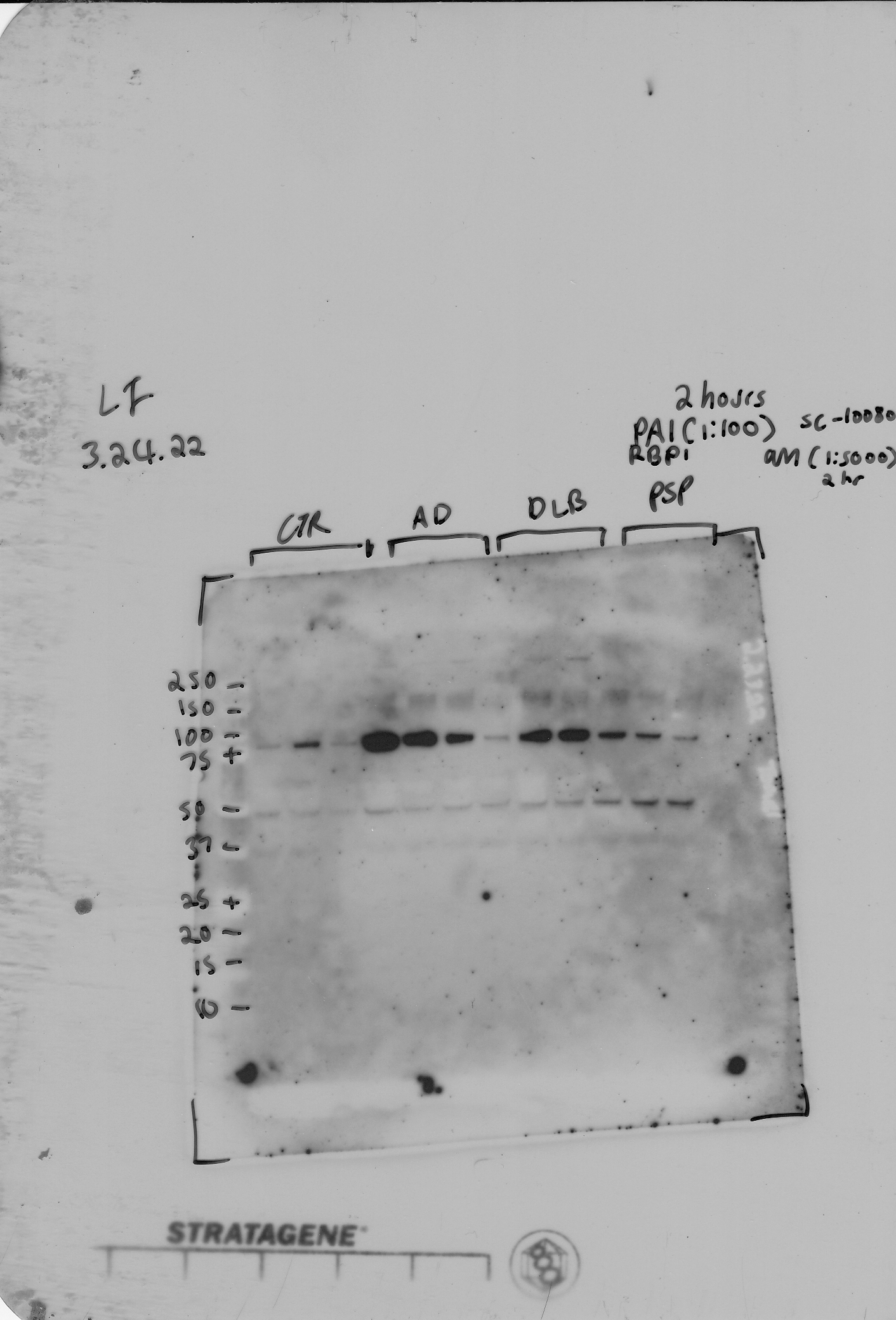

Supplement: Figure 7—source data 2. [file elife-98152-fig7-data2.zip › Figure 7- source data 2. Original westerns in Figure 7D/3.22.22 PAI-RBP1 TOMA1 membrane2 hrs_0001.tif]
