# Supplementary material for: SERBP1 interacts with PARP1 and is present in PARylation-dependent protein complexes regulating splicing, cell division, and ribosome biogenesis
Source: eLife. 2025 Feb 12;13:RP98152. doi: 10.7554/eLife.98152 (PMC11820137; doi:10.7554/eLife.98152)
Supplement: Figure 1—figure supplement 2—source data 1. [file elife-98152-fig1-figsupp2-data1.pdf]

Figure 1–figure supplement 2 source data 1. PDF containing original westerns for Figure 1–figure supplement 2A.

Puromycin:

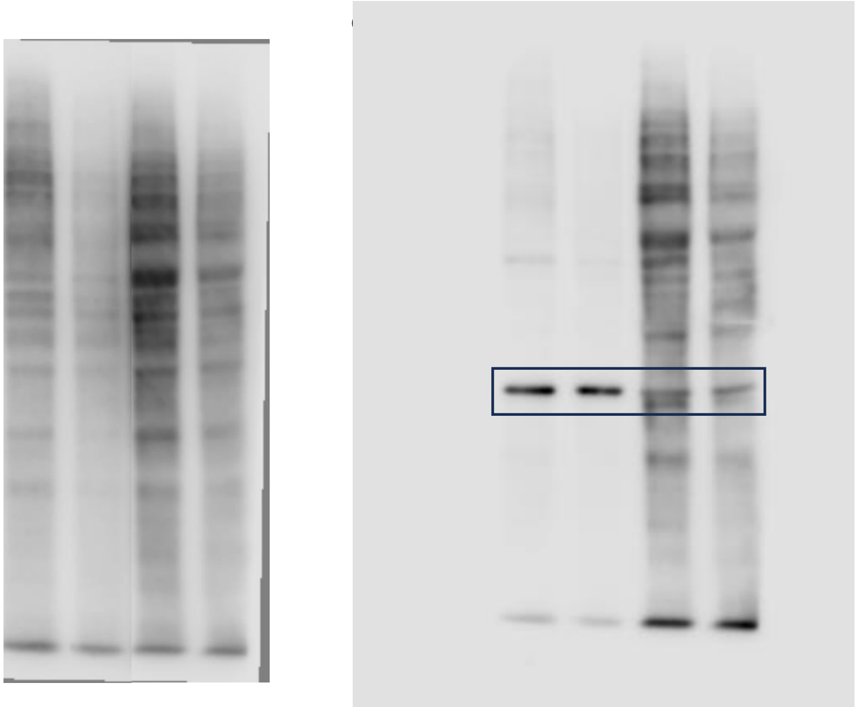

U251 SERBP1:

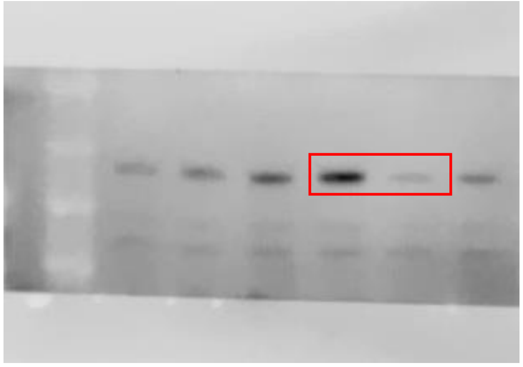

U251 β-Actin

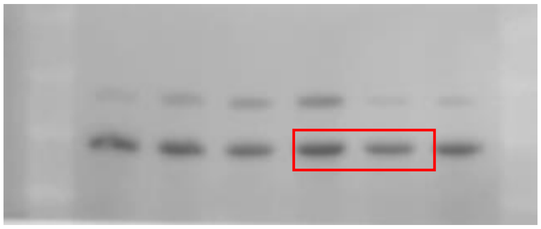

U343 SERBP1:

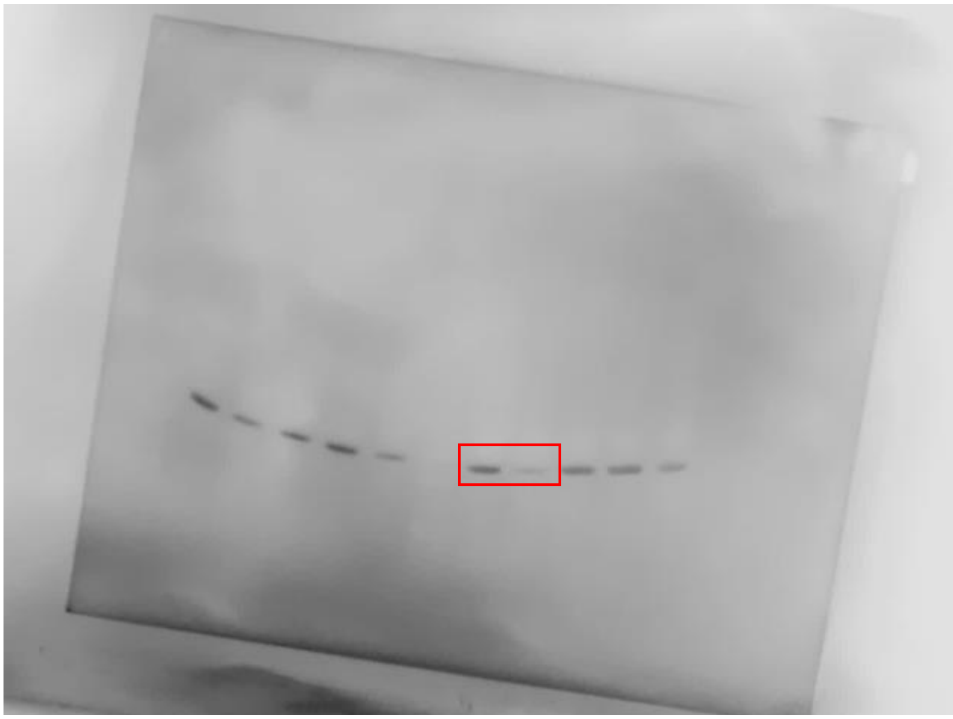

U343 β-Actin

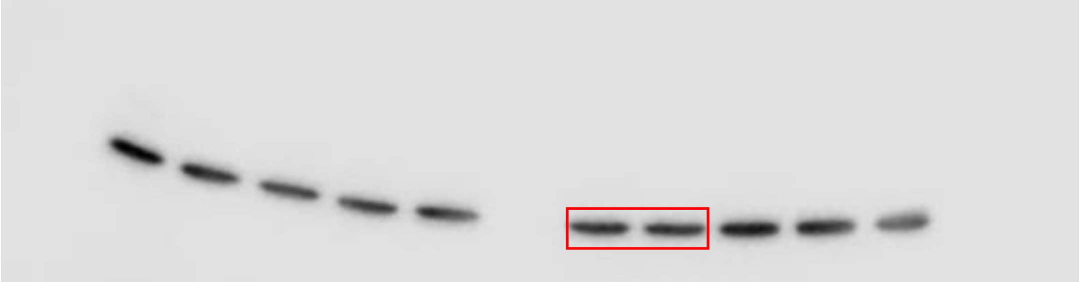

Supplementary Figure 2A: Translation level in U251 and U343 with or without SERBP1 knock down as determined by puromycin incorporation assay.
