# Supplementary material for: SERBP1 interacts with PARP1 and is present in PARylation-dependent protein complexes regulating splicing, cell division, and ribosome biogenesis
Source: eLife. 2025 Feb 12;13:RP98152. doi: 10.7554/eLife.98152 (PMC11820137; doi:10.7554/eLife.98152)
Supplement: Figure 2—source data 1. [file elife-98152-fig2-data1.pdf]

Figure 2-source data 1:PDF file containing original western blots for Figure 2B

U251 SERBP1:

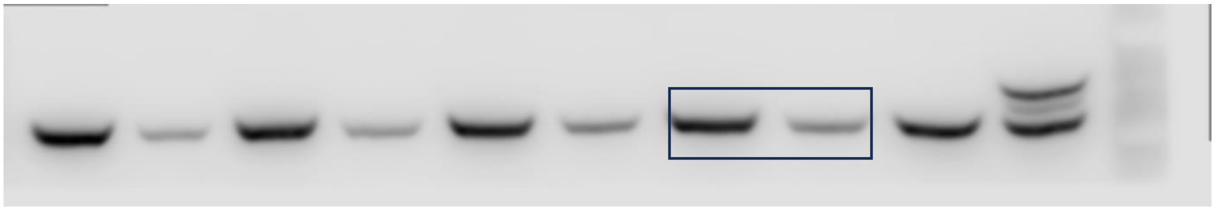

U251 b-Tubulin:

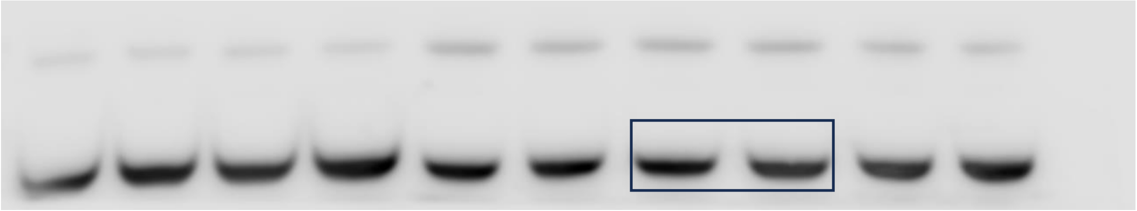

U343 SERBP1:

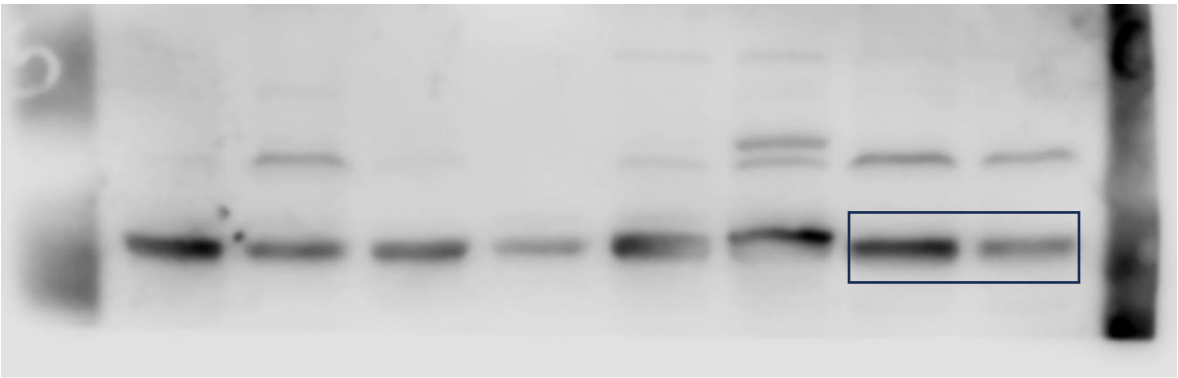

U343 b-Tubulin:

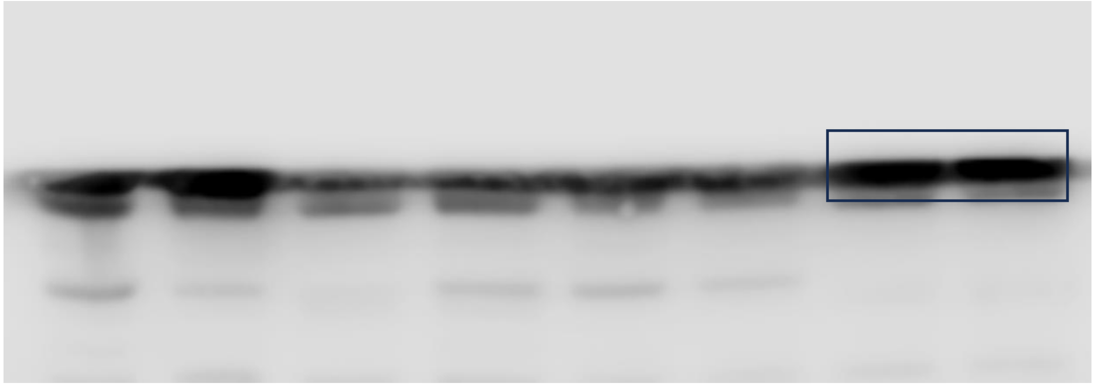

Figure 2B. Western blot showing SERBP1 knockdown in U251 and U343 cells.
