# Supplementary material for: SERBP1 interacts with PARP1 and is present in PARylation-dependent protein complexes regulating splicing, cell division, and ribosome biogenesis
Source: eLife. 2025 Feb 12;13:RP98152. doi: 10.7554/eLife.98152 (PMC11820137; doi:10.7554/eLife.98152)
Supplement: Figure 4—source data 1. [file elife-98152-fig4-data1.pdf]

Figure 4 - source data 1. PDF file containing original western blots for Figure 4A

PARP1:

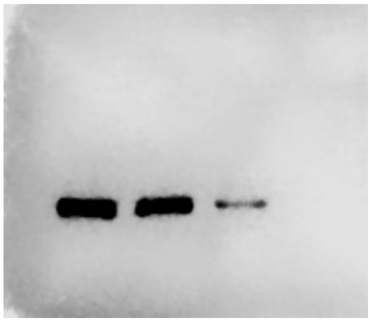

NCL:

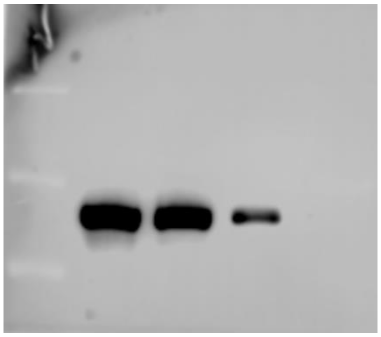

SYNCRIP:

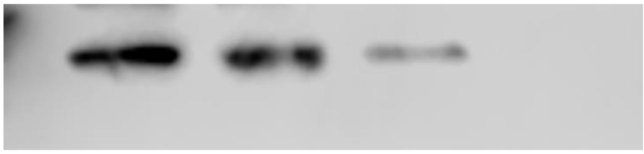

GAPDH:

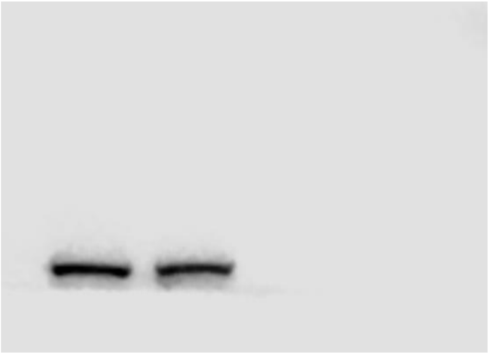

Figure 4A Results of IP-Western in U251 cells with control and anti-SERBP1 antibody confirm SERBP1 interaction with PARP1, NCL and SYNCRIP.
