# Supplementary material for: SERBP1 interacts with PARP1 and is present in PARylation-dependent protein complexes regulating splicing, cell division, and ribosome biogenesis
Source: eLife. 2025 Feb 12;13:RP98152. doi: 10.7554/eLife.98152 (PMC11820137; doi:10.7554/eLife.98152)
Supplement: Figure 4—source data 2. [file elife-98152-fig4-data2.pdf]

Figure 4-source data 2. PDF containing original silver staining gel and western blot for Figure 4E

Silver staining:

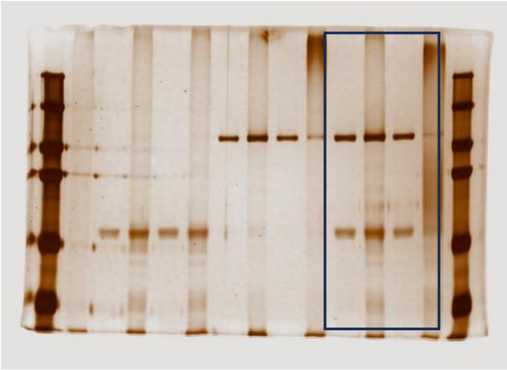

PAR:

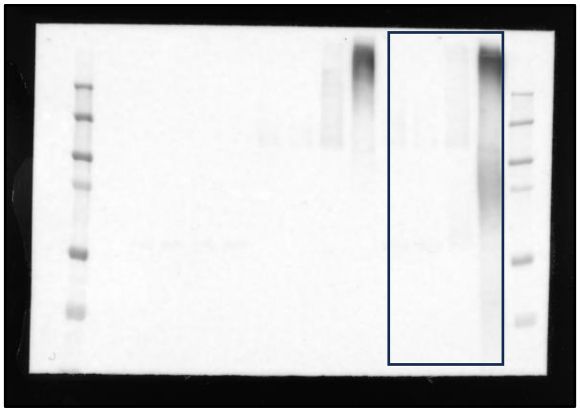

Figure 4E PARP1 ADP-ribosylates SERBP1 in vitro. Purified recombinant PARP1 and SERBP1 were combined in a reaction with or without sssDNA and NAD+. The reaction products were analyzed by SDS-PAGE with silver staining and Western blot for PAR.
