# Supplementary material for: SERBP1 interacts with PARP1 and is present in PARylation-dependent protein complexes regulating splicing, cell division, and ribosome biogenesis
Source: eLife. 2025 Feb 12;13:RP98152. doi: 10.7554/eLife.98152 (PMC11820137; doi:10.7554/eLife.98152)
Supplement: Figure 4—source data 3. [file elife-98152-fig4-data3.pdf]

Figure 4-source data 3. PDF file containing original westerns for Figure 4G

Poly-ADP-ribose:

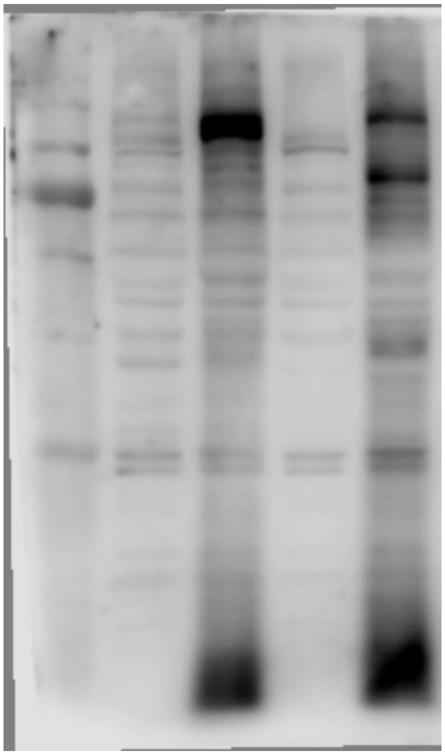

b-actin:

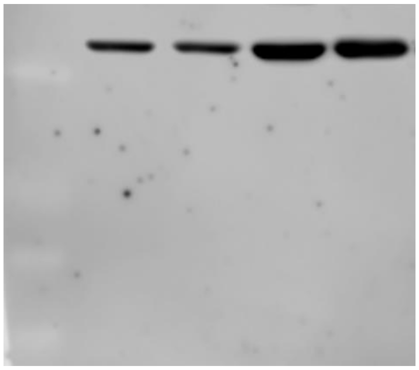

Figure 4G: Increase of PARylation levels in 293T and U251 GBM cells after H2O2 treatment.
