# Supplementary material for: SERBP1 interacts with PARP1 and is present in PARylation-dependent protein complexes regulating splicing, cell division, and ribosome biogenesis
Source: eLife. 2025 Feb 12;13:RP98152. doi: 10.7554/eLife.98152 (PMC11820137; doi:10.7554/eLife.98152)
Supplement: Figure 4—source data 4. [file elife-98152-fig4-data4.pdf]

Figure 4-source data 4. PDF file containing original westerns for Figure 4H

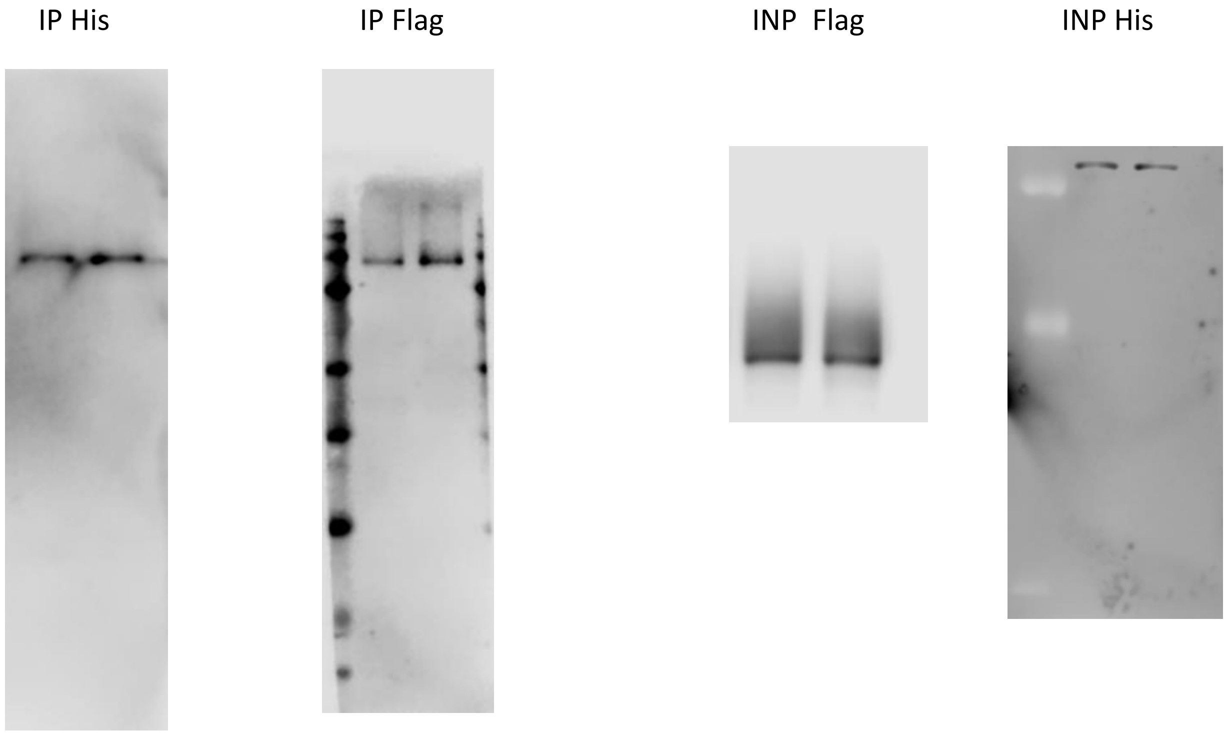

Figure 4H: SBP-SERBP1 pulldown in 293T cells shows increased SERBP1 association with PARP1 upon H2O2 treatment. SBP-SERBP1-His detected by His antibody. Flag-PARP1 detected by Flag antibody.
