# Supplementary material for: SERBP1 interacts with PARP1 and is present in PARylation-dependent protein complexes regulating splicing, cell division, and ribosome biogenesis
Source: eLife. 2025 Feb 12;13:RP98152. doi: 10.7554/eLife.98152 (PMC11820137; doi:10.7554/eLife.98152)
Supplement: Figure 4—source data 5. [file elife-98152-fig4-data5.pdf]

Figure 4-source data 5. PDF file containing original westerns for Figure 4I

Figure. 4I:

PARP1:

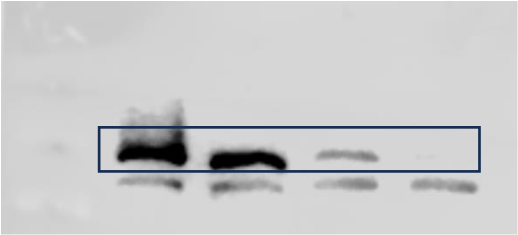

G3BP1:

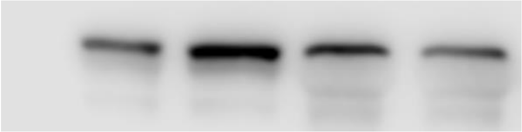

SERBP1:

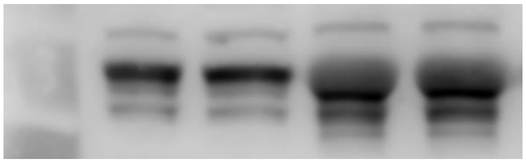

GAPDH:

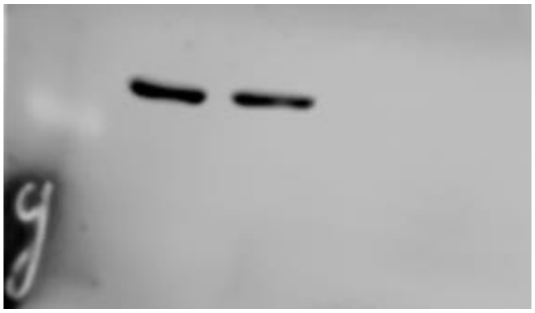

Figure 4I: PARP1 inhibitor PJ34 treatment repressed the interaction of SERBP1 with PARP1 and G3BP1.
