# Supplementary material for: SERBP1 interacts with PARP1 and is present in PARylation-dependent protein complexes regulating splicing, cell division, and ribosome biogenesis
Source: eLife. 2025 Feb 12;13:RP98152. doi: 10.7554/eLife.98152 (PMC11820137; doi:10.7554/eLife.98152)
Supplement: Figure 4—source data 6. [file elife-98152-fig4-data6.pdf]

Figure 4-source data 6. PDF file containing original westerns for Figure 4J

Poly-ADP-ribose:

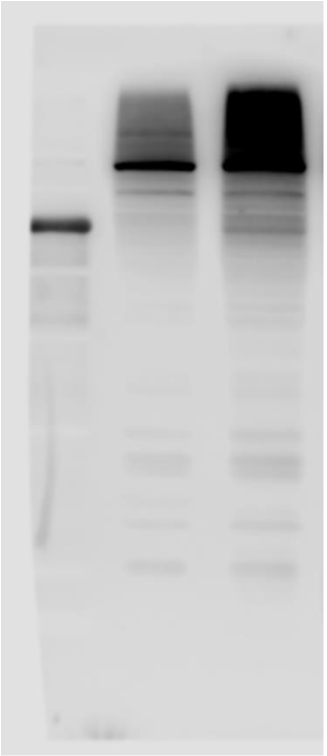

SERBP1:

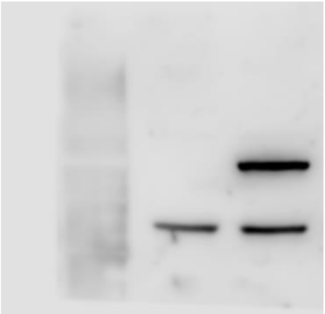

GAPDH:

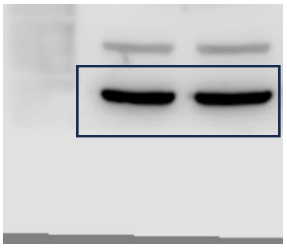

Figure 4J: SERBP1 transgenic expression (mGreen-SERBP1) in 293T cells increased the levels of PARylated proteins.
