# Supplementary material for: SERBP1 interacts with PARP1 and is present in PARylation-dependent protein complexes regulating splicing, cell division, and ribosome biogenesis
Source: eLife. 2025 Feb 12;13:RP98152. doi: 10.7554/eLife.98152 (PMC11820137; doi:10.7554/eLife.98152)
Supplement: Figure 4—figure supplement 1—source data 1. [file elife-98152-fig4-figsupp1-data1.pdf]

Figure 4- figure supplement 1- source data 1. PDF file containing western blots for Figure 4- figure supplement 1B.

Poly-ADP-ribose:

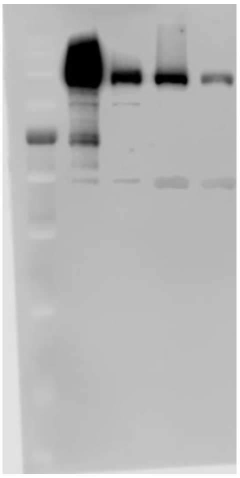

SERBP1:

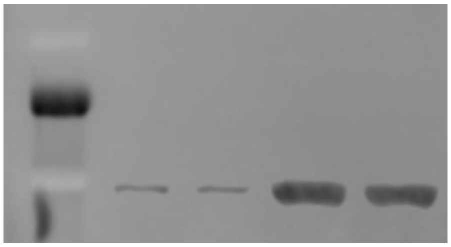

GAPDH:

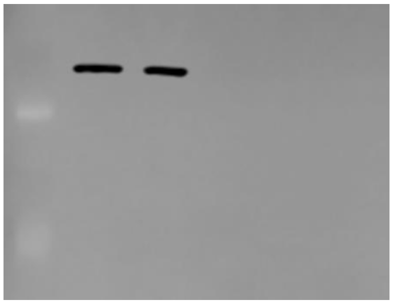

Figure 4- figure supplement 1B: Effect of PARylation/PAR binding on SERBP1 protein interactions. SERBP1 binding PAR proteins decreased upon PJ34 treatment.
