# Supplementary material for: SERBP1 interacts with PARP1 and is present in PARylation-dependent protein complexes regulating splicing, cell division, and ribosome biogenesis
Source: eLife. 2025 Feb 12;13:RP98152. doi: 10.7554/eLife.98152 (PMC11820137; doi:10.7554/eLife.98152)

Figure 7 - source data 1. PDF containing original westerns for Figure 7D, indicating bands and treatments.

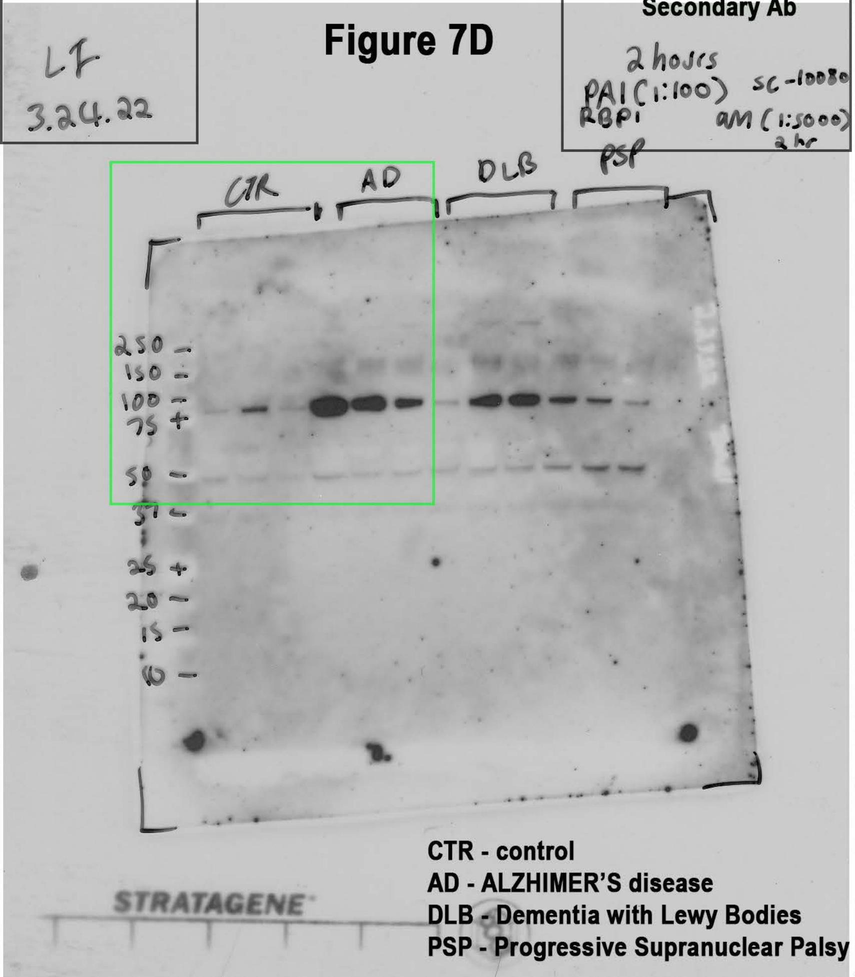

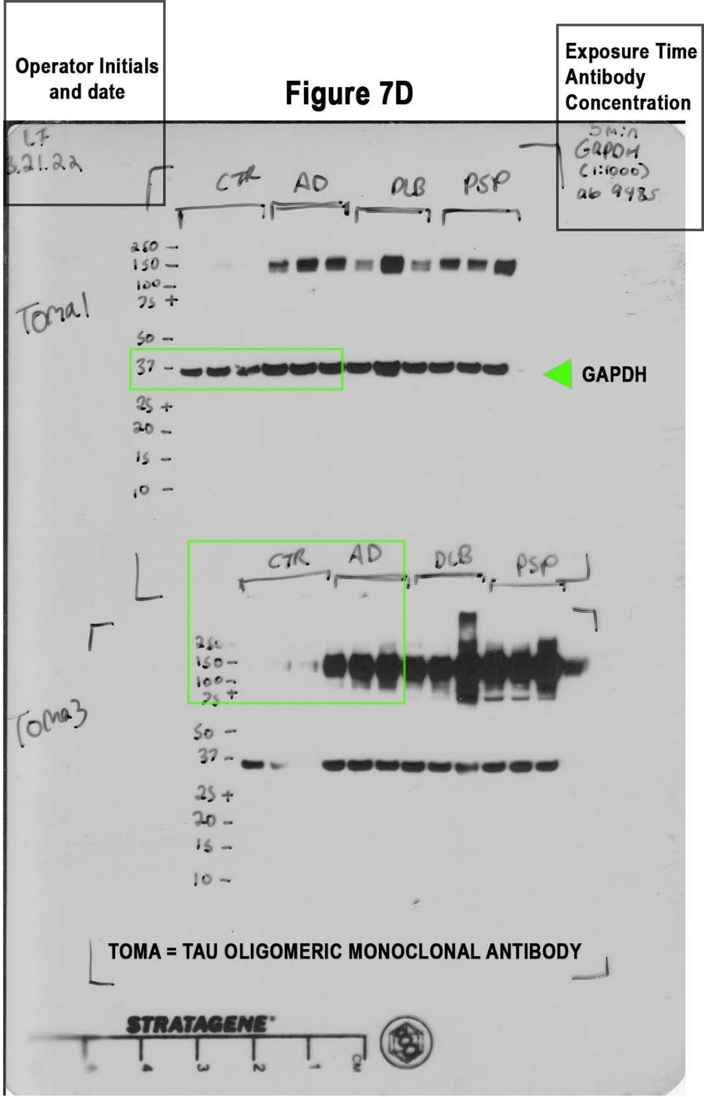

Supplement: Figure 7—source data 1. [file elife-98152-fig7-data1.pdf]
